# The novel GlcNAc 6-phosphate dehydratase NagS governs a metabolic checkpoint that controls nutrient signaling in *Streptomyces*

**DOI:** 10.1371/journal.pbio.3003514

**Published:** 2025-11-25

**Authors:** Chao Li, Mia Urem, Ioli Kotsogianni, Josephine Lau, Chao Du, Somayah S. Elsayed, Nathaniel I. Martin, Iain W. McNae, Patrick Voskamp, Christoph Mayer, Sébastien Rigali, Navraj Pannu, Jan Pieter Abrahams, Lennart Schada von Borzyskowski, Gilles P. van Wezel

**Affiliations:** 1 Institute of Biology, Leiden University, Leiden, The Netherlands; 2 School of Life Sciences, Anhui Agricultural University, Hefei, Anhui, China; 3 Department of Medical Microbiology, Leiden University Medical Center, Leiden, The Netherlands; 4 Biological Chemistry Group, Institute of Biology, Leiden University, Leiden, The Netherlands; 5 School of Biological Sciences, University of Edinburgh, Edinburgh, United Kingdom; 6 Leiden Institute of Chemistry, Leiden University, Leiden, The Netherlands; 7 Institute for Microbiology and Biotechnology, University of Tübingen, Tübingen, Germany; 8 InBioS—Center for Protein Engineering, University of Liège, Liège, Belgium; 9 Biozentrum, Basel University, Basel, Switzerland; 10 Paul Scherrer Institute, Villigen, Switzerland; 11 Department of Microbial Ecology, Netherlands Institute of Ecology (NIOO-KNAW), Wageningen, The Netherlands; Johns Hopkins University School of Medicine, UNITED STATES OF AMERICA

## Abstract

*Streptomyces* bacteria are renowned for their multicellular lifestyle and as Nature’s medicine makers, producing the majority of the clinical antibiotics. A landmark event during early development is the lytic dismantling of the substrate mycelium. Degradation of the hyphal cell-wall leads to the accumulation of *N*-acetylglucosamine (GlcNAc) in the colonies, which is a metabolic checkpoint during the onset of development and antibiotic production. Here, we show that GlcNAc sensing requires a toxicity pathway dependent on the enzyme GlcNAc-6P dehydratase (NagS). Dehydration of GlcNAc-6P by NagS to 6P-chromogen I is an unprecedented reaction in central metabolism that is highly conserved in – and limited to – the *Streptomycetaceae*. 6P-chromogen I is metabolized into a structural analogue of ribose by a promiscuous activity of GlcNAc-6P deacetylase NagA. Toxicity is relieved by supplementing the growth media with ribose. Structure-function analysis of NagS not only highlighted key residues in the active site of the enzyme in interaction with its substrate GlcNAc-6P, but also revealed 6-phosphogluconate as its catalytic inhibitor. Our work uncovers a conserved metabolic toxicity pathway in *Streptomyces* that revolves around a novel enzyme that plays a key role in nutrient signaling.

## Abstract 2

N-acetylglucosamine (GlcNAc) accumulation acts as a key metabolic signal in Streptomyces, but how it triggers developmental responses is unclear. This study shows that the novel enzyme NagS dehydrates GlcNAc-6P into a reactive intermediate, triggering a toxicity-based checkpoint that governs nutrient signalling in Streptomyces.

## Introduction

Streptomycetes are Gram-positive filamentous bacteria with a complex multicellular life cycle [1]. They are known as Nature’s medicine makers, producing two-thirds of all clinical antibiotics, as well as many other natural products with anticancer, anthelmintic, antifungal, or immunosuppressant bioactivity [2–4]. *Streptomyces* development has been studied extensively. Early development is controlled by the *bld* (bald) genes, named so for the lack of the fluffy white aerial hyphae in *bld* mutants [5,6]. A number of the Bld proteins form a signaling cascade that eventually leads to the accumulation of the lanthipeptide SapB, a hydrophobic morphogen that promotes aerial hyphae formation [7,8]. Sporulation is controlled by the *whi* (white) genes, mutants of which fail to produce gray-pigmented spores [9,10]. Activation of antibiotic production is coordinated with the onset of development [11,12]. An important late cell-cycle checkpoint is the activation of sporulation-specific cell division and its coordination with DNA segregation. A key event is the recruitment of the cell division protein FtsZ to future septum sites by SsgB [13], the expression of which in turn is controlled by the Whi regulatory proteins [14] and the SOS response [15].

A landmark event during the earliest stage of development and antibiotic production in streptomycetes is the lytic degradation of the cell-wall, a remarkable example of programmed cell death (PCD) whereby a large part of the vegetative or substrate mycelium is degraded to provide nutrients to produce aerial mycelium and spores in a nutrient-depleted environment [16,17]. DNA-degrading molecules, such as prodiginines in *Streptomyces coelicolor*, promote the killing of the substrate hyphae in the center of the colony, providing nutrients and allowing the colony to expand prior to sporulation [17]. A major metabolic signal during early development is the accumulation in the colonies of *N*-acetylglucosamine (GlcNAc), which is a building block of the cell-wall peptidoglycan. GlcNAc forms a nutrient status-dependent switch, whereby it promotes growth under rich nutritional conditions (*feast*), thereby repressing development and antibiotic production. Conversely, GlcNAc activates development under nutrient-limiting conditions (*famine*) [18].

The GlcNAc-mediated activation of antibiotic production revolves around DasR, a global nutrient sensory regulator that controls the uptake and metabolism of GlcNAc [18,19], and a master regulator that controls all biosynthetic gene clusters (BGCs) for antibiotic and siderophore biosynthesis in *S. coelicolor* [20–22]. This discovery is also important in terms of the search for new antibiotics. Many of the BGCs are silent in the laboratory, and to uncover the hidden chemical space, we need to understand the triggers and cues that activate these BGCs [23,24]. Many regulators respond to small metabolites that act as ligands that affect their DNA binding capacity, and hence a better understanding of the correlation between primary and secondary metabolism is of key importance [25,26].

GlcNAc is imported by the phosphoenolpyruvate phosphotransferase system (PTS) as GlcNAc-6P [27]. NagA deacetylates GlcNAc-6P to form glucosamine-6-phosphate (GlcN-6P), which is then deaminated by NagB to produce fructose-6P (Fru-6P). GlcNAc-6P and GlcN-6P allosterically induce the release of DasR from its recognition sites, thereby attenuating its transcriptional inhibition and activating antibiotic production (S1 Fig) [18,22]. Deletion of *dasR* leads to the same phenotype as adding GlcNAc, with accelerated development and antibiotic production on minimal media and vegetative arrest on rich media, suggesting that DasR controls both development and antibiotic production. However, while DasR controls GlcNAc metabolism and all antibiotic pathways in *S. coelicolor*, it does not directly bind to developmental genes, except for the *bldK* operon, which encodes an oligopeptide transporter involved in iron acquisition [21]. This is in contrast to the regulon of the developmental master regulator BldD, which represses a vast number of developmental genes [28]. While DasR senses amino sugar levels, BldD binding depends on the modified nucleotide cyclic-di-GMP [29], underlining the importance of metabolic signaling during development of streptomycetes.

*Streptomyces coelicolor nagB* mutants are sensitive to both GlcN and GlcNAc; this phenomenon was exploited to obtain spontaneous suppressor mutants that allowed *nagB* mutants to grow in the presence of GlcNAc or GlcN, in search of new genes involved in amino sugar sensing [30]. While deletion of *nagA* relieves the toxicity of both GlcN and GlcNAc to *nagB* mutants, genes were also identified that relate specifically to GlcN or GlcNAc. Mutants in the regulatory gene *rokL6* relieve GlcN toxicity by enhancing the expression of MFS transporter SCO1448 [31]. Conversely, inactivation of the gene for SCO4393, for an unknown enzyme with a sugar isomerase (SIS) domain, specifically relieves toxicity of GlcNAc.

In this work, we show that SCO4393 plays a key role in GlcNAc sensing during early stages of development and controls a set of metabolic reactions that explain GlcNAc toxicity. The enzyme was renamed to NagS, for *N*-acetylglucosamine sensitivity. NagS remarkably acts as an *N*-acetylglucosamine-6-phosphate dehydratase, a reaction that was previously unknown in central metabolism. NagA deacetylates the product of NagS to form a ribose-like compound that is key to GlcNAc toxicity. X-ray crystallography provided key insights into the substrate binding site of NagS, and helped identifying its inhibitor 6-phosphogluconate. Our data provide new insights into a unique metabolic pathway within streptomycetes that influences nutrient-mediated control of morphological and chemical differentiation.

## Results

### NagS is required for GlcNAc toxicity and amino sugar sensing

Mutants of *S. coelicolor* that lack *nagB* fail to grow on minimal media (MM) supplemented with GlcNAc. In order to obtain mutants that are affected in GlcNAc sensing and nutrient control of development and antibiotic production, mutants were analyzed that we previously obtained in a suppressor screen, selecting for spontaneous mutants of *S. coelicolor nagB* mutants that survive specifically on GlcNAc [30]. Sequencing of suppressor mutant SMA11 revealed a single nucleotide polymorphism (SNP) at nucleotide position 535 of SCO4393 (G to A substitution), leading to a nonsilent change from aspartate to asparagine (D179N) in the predicted gene product. SCO4393 contains a so-called SIS domain [32], which spans the entire protein (see below). As SCO4393 was shown to be involved in *N*-acetylglucosamine sensitivity, this gene was renamed *nagS*.

**Fig 1:**
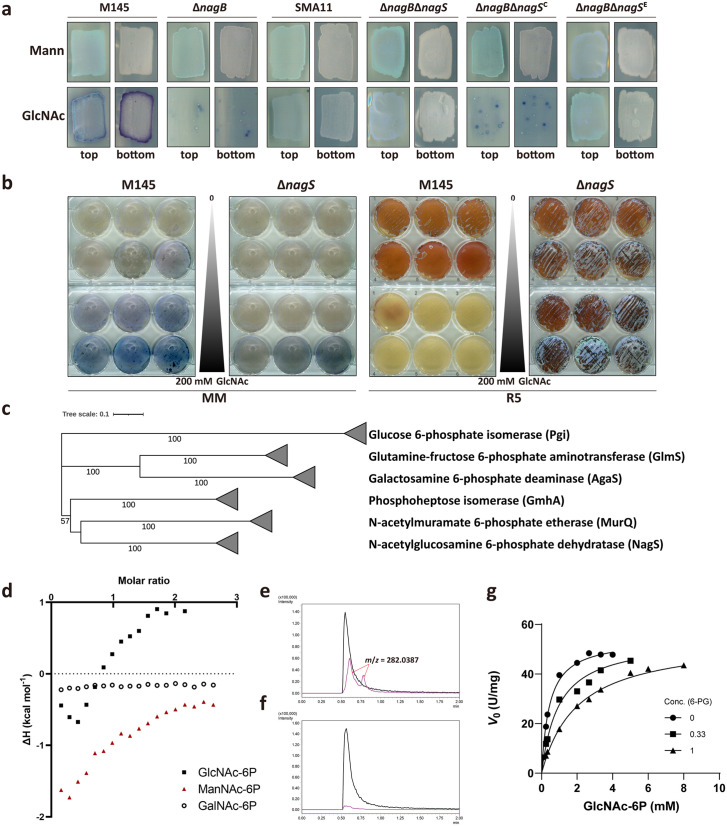
NagS is a novel GlcNAc-6P dehydratase. **(a)** Sensitivity of *S. coelicolor* mutants to GlcNAc. Spores (5 × 10^5^ CFU) of *S. coelicolor* M145 and its mutant derivatives ∆*nagB*, SMA11, ∆*nagB*∆*nagS*, ∆*nagB*∆*nagS*^C^ (∆*nagB*∆*nagS* expressing *nagS*) and ∆*nagB*∆*nagS*^E^ (∆*nagB*∆*nagS* with empty plasmid pSET152) were streaked on MM agar plates with 1% mannitol (Mann) and 1% mannitol plus 10 mM GlcNAc (GlcNAc). Strains were cultured for 72 h at 30 °C. Note that *nagB* mutants can grow in the presence of 10 mM GlcNAc only when SCO4393 (*nagS)* has been mutated (suppressor SMA11) or deleted. **(b)** NagS and its role in GlcNAc sensing. Spores of M145 and ∆*nagS* were plated on MM and R5 with 0, 0.001, 0.01, 0.1, 1, 5, 10, 20, 50, 100, 150, and 200 mM GlcNAc. Note that *nagS* mutants hardly respond to GlcNAc. **(c)** Phylogenetic tree of several different types of sugar isomerase (SIS) domain enzymes in bacteria, including NagS, Glucose-6P isomerase (Pgi), Phosphoheptose isomerase (GmhA), *N*-acetylmuramic acid-6-phosphate etherase (MurQ), Glutamine-fructose-6-phosphate aminotransferase (GlmS), and putative D-galactosamine-6-phosphate deaminase (Agas). The phylogenetic tree was made by MEGA11 (Neighbour-joining method) and built based on the alignment of the amino acid sequences. **(d)** ITC analysis of NagS with *N*-acetyl-6-phosphate amino sugar metabolites, namely GlcNAc-6P, ManNAc-6P, and GalNAc-6P. Both GlcNAc-6P and ManNAc-6P are bound well by NagS, while the enzyme did not bind to GalNAc-6P. (**e and f**) confirmation of NagS products by LC-MS. Extracted ion chromatograms for GlcNAc-6-P (black trace) and its dehydrated product (pink trace) in the enzymatic reaction mixture of GlcNAc-6-P with either active NagS (e) or heat-inactivated NagS **(f)**. Peaks relating to the product (*m*/*z* = 282.0387) are indicated by red arrows. **(g)** Kinetics of NagS in the presence of 6-PG (0, 0.33, 1 mM). The *V*_0_ data used in d were plotted against the substrate concentration, and each assay was performed in triplicate and expressed as a mean ± standard error. The data underlying this Figure can be found in S1 Data.

To study the role of NagS in GlcNAc metabolism and nutrient sensing, we created a *nagB*-*nagS* double mutant. While the parental strain *S. coelicolor* M145 grew normally on MM with 1% (w/v) mannitol and 10 mM GlcNAc, the *nagB* mutant was unable to grow under these conditions. Conversely, both the suppressor mutant SMA11 and the ∆*nagB*∆*nagS* mutant grew well in the presence of GlcNAc. To ascertain that the deletion of *nagS* was the sole cause of the observed phenotypes described, we genetically complemented the ∆*nagB*∆*nagS* mutant with a native copy of *nagS*. Expression of *nagS* restored sensitivity of the mutants to GlcNAc (Fig 1a). These data strongly suggest that the deletion of *nagS* was the sole cause of the restored growth on GlcNAc in *nagB* mutants.

We then wondered if NagS might play a role in GlcNAc-dependent control of development and specialized metabolism. To investigate this, *S. coelicolor* was cultured on MM and nutrient-rich R5 agar plates with increasing concentrations of GlcNAc. Expectedly, GlcNAc enhanced development and antibiotic production of wild-type *S. coelicolor* on MM and inhibited these processes on R5. In contrast, the *nagS* mutant showed normal sporulation on R5 agar even at GlcNAc concentrations up to 200 mM, and the mutant failed to show the typical enhanced antibiotic production when MM was supplemented with GlcNAc (Fig 1b). In other words, GlcNAc sensing had been lost in *nagS* mutants. To further investigate this, we also looked at siderophore production, as GlcNAc also efficiently blocks siderophore biosynthesis in *S. coelicolor* M145 on R5 agar plates [18]. We therefore wondered if the biosynthesis of siderophores would still be subjected to catabolite repression by GlcNAc. Interestingly, *nagS* null mutants had lost the ability to suppress siderophore production on R5 with added GlcNAc (S2 Fig). Taken together, these observations indicate that NagS plays a pivotal role in amino sugar sensing in streptomycetes, highlighting its essential function in the nutrient-dependent regulation of differentiation depending on the extracellular concentration of GlcNAc.

To assess, suppressor mutants can still be obtained in the *S. coelicolor nagB* null mutant when NagS or NagA levels are enhanced, we expressed either *nagA* or *nagS* using the strong and constitutive *ermE* promoter (S3a Fig). Constitutive expression of *nagS* in ∆*nagB* reduced the appearance of suppressor mutants by an order of magnitude when the strains were grown on GlcNAc, while over-expression of *nagA* prevented the emergence of suppressor mutants even at 10^8^ colony-forming units (CFU). A control experiment showed that introducing P_*ermE*_-*nagA* in the *nagBS* double mutant or P_*ermE*_-*nagS* in the *nagAB* double mutant did not prevent growth on GlcNAc (S3b Fig). These experiments underline that both NagA and NagS mediate GlcNAc toxicity. Since GlcN-6P, the product of NagA, is not a catalytic substrate of NagS, these data suggest that NagA might have a second function by acting on the product of NagS, leading to the formation of toxic compounds derived from GlcNAc (this is worked out further below).

### Phylogenetic analysis of NagS

**Fig 2:**
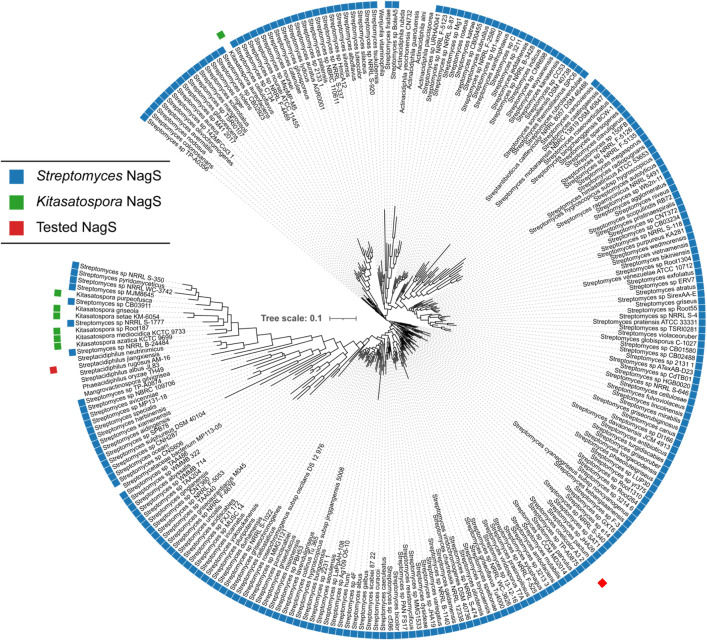
Phylogenomic analysis of NagS. Phylogenetic tree of NagS proteins in the StringDB database. The branches are annotated with the strain names in which the orthologues occur. *Streptomyces* and *Kitasatospora* strains are denoted by blue and green markers, respectively. NagS was exclusively found in members of the *Streptomycetaceae.* The NagS proteins from *S. coelicolor* and *Streptacidiphilus jiangxiensis*, whose GlcNAc-6P dehydratase activity was verified by enzyme assays in vitro, are highlighted with a red marker.

To see how NagS is distributed across different phyla, we performed phylogenetic analysis. NagS contains a conserved SIS domain [32], predicted to span almost the entire length of the protein. SIS domains are typically found in phosphosugar-binding proteins, including *N*-acetylmuramic acid-6-phosphate etherase (MurQ) and glutamine-fructose-6-phosphate aminotransferase (GlmS) [33–35]. Phylogenetic analysis of NagS homologs together with other SIS domain proteins revealed that NagS is related to MurQ but forms a well-defined and separate clade in the phylogenetic tree (Fig 1c). Phylogenomic analysis of all NagS proteins in StringDB revealed that NagS is conserved in all streptomycetes except for two marine-derived species (CNH-099 and CNQ-509 [36,37]) in the target database (StringDB v12.0) [38]. Orthologues of NagS were identified exclusively in the *Streptomycetacea*e, namely in *Streptomyces*, *Kitasatospora*, *Streptacidiphilus*, *Actinacidiphila,* and a few rare genera with a single representative (Figs 2 and S4a; S4 Table). Their overall amino acid identity was 58.7% or more. Outside the *Streptomycetaceae* more distant homologs were identified, with an amino acid identity of around 30%. Enzyme assays showed that these enzymes do not have NagS activity and have therefore not been included in the phylogenetic tree (see below). Gene synteny analysis showed that the genomic region around *nagS* is also conserved, whereby *nagS* is invariably located adjacent to and divergently expressed from the iron master regulatory gene *dmdR1* (SCO4394) [39,40]. Importantly, this linkage between *nagS*-*dmdR1* exists in all *Streptomycetaceae* (S4b Fig).

### GlcNAc-6P and ManNAc-6P are substrates of NagS

In order to identify the substrate of NagS, we first expressed *S. coelicolor* NagS-His_6_ in *Escherichia coli* and purified the protein to homogeneity using Ni-affinity chromatography. Size-exclusion chromatography revealed it to be a dimer (S7 Table, S5 Fig). Isothermal titration calorimetry (ITC) was performed with Glc-6P, Fru-6P, GlcN-6P, GlcNAc, GlcNAc-1P, and GlcNAc-6P. Of these, only GlcNAc-6P showed significant binding to NagS (S6a Fig), suggesting that both the *N*-acetyl moiety and the C6 phosphate group are important for NagS binding. We then tested binding of GlcNAc-6P and its epimers *N*-acetylmannosamine-6-phosphate (ManNAc-6P) and *N*-acetylgalactosamine-6-phosphate (GalNAc-6P) as ligands. This revealed that both GlcNAc-6P and ManNAc-6P were bound by NagS, while GalNAc-6P was not (Fig 1d). Notably, a change in absorbance at 230 nm was detected when either 1 mM GlcNAc-6P or ManNAc-6P was incubated with purified NagS at 30 °C (S6b–S6d Fig), suggesting that NagS converted both compounds in vitro into hitherto unknown products. This is consistent with the ITC result that did not display a simple binding isotherm, as both GlcNAc-6P and ManNAc-6P are substrates of NagS, and substrate turnover is therefore expected to occur during the measurement.

### NagS is an *N*-acetylglucosamine-6-phosphate dehydratase

The product formed from the conversion of GlcNAc-6P by NagS was identified based on NMR and Liquid Chromatography–Mass Spectrometry (LC–MS). Following in vitro reactions using GlcNAc-6P as the substrate, new peaks were detected in the ^1^H NMR spectrum specifically for the reaction with active NagS, which were not seen following incubation with heat-inactivated enzyme (S7a Fig). The chemical shifts of the reaction product pointed at the formation of phosphorylated Chromogen I, the 2,3-dehydro derivative of GlcNAc (S5 Table, S8 Fig) [41]. 6P-Chromogen I (2-acetamido-2,3-dideoxy-6-phosphate-D-erythro-hex-2-enofuranose; designated **1**) exists as a mixture of its α and β anomers. This was further confirmed by LC–MS analysis, which showed the appearance of two peaks in the reaction mixture containing the active NagS (Fig 1e, 1f). The HRESIMS spectrum of the peaks (S7b Fig) established a molecular formula of C_8_H_14_NO_8_P (*m/z* 282.0387), which is consistent with the structure identified by NMR for **1** (S6 Table). NagS also acts on the other substrate, ManNAc-6P, resulting in its dehydration, although the NMR signals corresponding to **1** were relatively lower in intensity as compared to the reaction with GlcNAc-6P (S7c Fig). Kinetics demonstrated a markedly higher catalytic efficiency of NagS for GlcNAc-6P, with *k*_cat_ and *K*_m_ values of 24.67 s^−1^ and 0.45 mM, respectively, which is approximately 40 times greater than that for ManNAc-6P, measured at 0.90 s^−1^ and 0.68 mM, respectively (Table 1 and S7e, S7f Fig). Taken together, our data show that NagS dehydrates both GlcNAc-6P and ManNAc-6P to produce **1** (S7d Fig). NagS and its homologs constitute a completely novel family of GlcNAc-6P dehydratases within the SIS domain superfamily.

**Table 1:** Kinetic parameters for *S. coelicolor* NagS and NagA.

Enzymes^*^	Substrates	*K*_m_ (mM)	*k*_cat_ (s^−1^)	*k*_cat_/*K*_m_ (M^−1^·s^−1^)
NagS	GlcNAc-6P	0.45 ± 0.03	24.67 ± 0.38	5.48 × 10^4^
NagS	ManNAc-6P	0.68 ± 0.05	0.90 ± 0.02	1.32 × 10^3^
NagS-H53A^#^	GlcNAc-6P	–	–	–
NagS-S54A	GlcNAc-6P	0.85 ± 0.09	2.70 ± 0.09	3.30 × 10^3^
NagS-R64A^#^	GlcNAc-6P	–	–	–
NagS-S91A	GlcNAc-6P	0.56 ± 0.04	0.78 ± 0.01	1.38 × 10^3^
NagS-E94A^#^	GlcNAc-6P	–	–	–
NagS-S119A	GlcNAc-6P	2.06 ± 0.27	3.83 ± 0.23	1.86 × 10^3^
NagS-S121A	GlcNAc-6P	1.19 ± 0.09	10.49 ± 0.28	8.84 × 10^3^
NagS-D179A^#^	GlcNAc-6P	–	–	–
NagS-N228A	GlcNAc-6P	1.77 ± 0.28	0.47 ± 0.03	263.30
NagA	GlcNAc-6P	1.59 ± 0.21	159.61 ± 9.02	1.01 × 10^5^
NagA	ManNAc-6P	2.40 ± 0.44	14.56 ± 1.10	6.06 × 10^3^
NagA	GalNAc-6P	2.84 ± 0.41	29.17 ± 2.31	1.03 × 10^4^

* Each assay was carried out in triplicate and is expressed as mean ± standard error.

# “–“ indicates that no reaction was observed.

### *N*-acetylglucosamine-6-phosphate dehydratase activity of NagS orthologs

Next, we tested SIS domain proteins with lower homology to NagS, namely proteins from *Streptacidiphilus jiangxiensis* (TrEMBL A0A1H7F721), *Clostridium amylolyticum* (TrEMBL A0A1M6IM34), *Paenibacillus selenitireducens* (TrEMBL A0A1T2XKX6), and *Acidothermus cellulolyticus* (TrEMBL A0LSD9). The amino acid identities with *S. coelicolor* NagS are 65.5%, 33.2%, 25.7%, and 35.7%, respectively (S9a Fig). The enzyme assays showed that only the protein from *S. jiangxiensis* exhibited significant activity, and thus qualifies as a *bona fide* NagS (designated NagS^Scjia^). The ortholog from *C. amylolyticum* showed negligible activity, while the other enzymes failed to catalyze the dehydration of GlcNAc-6P (S9b–S9e Fig). To verify these data, we investigated whether expression of the different genes could genetically complement *S. coelicolor* ∆*nagB*∆*nagS* by restoring sensitivity to GlcNAc on MM agar plates. Expectedly, introduction of a clone expressing *nagS*^Scjia^ in *S. coelicolor* ∆*nagB*∆*nagS* restored sensitivity of the strain to 10 mM GlcNAc (S9f Fig), which confirms that NagS from *S. jiangxiensis* indeed acts as a real NagS enzyme in vivo. Conversely, introduction of constructs expressing any of the other genes in the *nagB-nagS* double mutant failed to restore GlcNAc sensitivity (S9f Fig). Therefore, it is likely that the more distant homologs from species outside the family of *Streptomycetacea* cannot use GlcNAc-6P as a substrate.

### 6-phosphogluconate is a competitive inhibitor of NagS

Next, we sought to identify potential other interaction partners of NagS to better understand its role in *S. coelicolor* carbon metabolism. Since 6-phosphogluconate (6-PG) structurally resembles linear GlcNAc-6P (S10a Fig), we assessed the interaction between 6-PG and NagS using thermal denaturation assays. Interestingly, the addition of 2 mM 6-PG shifted the *T*_*m*_ of NagS from 46.45 °C ± 0.4 °C and 58.0 °C ± 0.5 °C, to 48.47 °C ± 0.28 °C and 58.33 °C ± 0.29 °C; while the addition of 5 and 10 mM 6-PG shifted the *T*_*m*_ from two separate peaks to a single merged peak with of *T*_m_ at 53.08 °C ± 0.03 °C at 10 mM, when compared against the NagS control (S10b Fig). This suggests that 6-PG may bind to NagS. 6-PG was not metabolized when incubated with NagS (S10c Fig), but instead it effectively inhibited the conversion of GlcNAc-6P (S10d Fig). Further kinetic analysis revealed that while the *V*_max_ remained constant, the *K*_m_ values for GlcNAc-6P increased with rising concentrations of 6-PG, observed at 0.33 and 1 mM (Figs 1g and S10e). The inhibition constant (*K*_i_) for 6-PG was determined to be 0.28 ± 0.03 mM, supporting its role as a competitive inhibitor. Additionally, the sensitivity of ∆*nagB* to GlcNAc was mitigated by supplementation with exogenous D-gluconate (S10f Fig).

### The crystal structure of NagS

**Fig 3:**
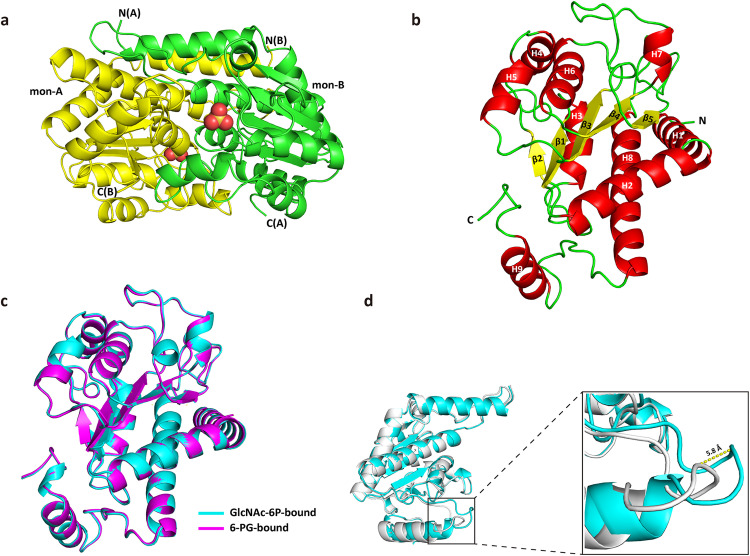
Crystal structure of NagS. **(a)** Dimeric structure of NagS determined by X-ray crystallography. The individual monomers (mon-A and mon-B) of the dimer are shown in green and yellow, with the N- and C- terminus indicated. Sulphates are shown in spheres. **(b)** Secondary structure of monomeric NagS. α-helices are shown in red, while β-sheets are shown in yellow and loops are shown in green. Monomer of NagS contains nine α-helices, H1-H9, and five β-sheets, β1-β5, as indicated. **(c)** Alignment of the secondary structure of GlcNAc-6P-bound NagS monomer (cyan) with that of monomer NagS in complex with 6-PG (magenta), with an RMS deviation of 0.17 for all atoms. **(d)** Alignment of the secondary structure of NagS apo monomer (white) with that of monomer NagS in complex with GlcNAc-6P (cyan), with an RMS deviation of 0.22 (200 to 200 atoms). The significant change of the loop (residues 226-232) is shown in the red box and zoomed in. The loop of NagS in complex with GlcNAc-6P shows a 5.8 Å shift as indicated by the yellow dotted line.

The atomic structures of apo-NagS and its complexes with GlcNAc-6P and 6-PG were determined by X-ray crystallography to resolutions of 2.3, 2.6, and 1.7 Å, respectively (S3 Table). The structures revealed that the biologically active dimer of NagS interacts along a crystallographic 2-fold axis with C2 symmetry (Fig 3a). Two identical substrate binding cavities are located at the dimeric interface (S11a Fig). This dimer interface, which sandwiched the substrate, is stabilized by evolutionarily conserved salt bridges and hydrogen bonds. Structurally, each monomer adopts an α/β configuration with a three-layered αβα sandwich architecture, featuring a standard parallel β-sheet surrounded by nine α-helices. The central β-sheet consists of five strands (β1–β5), contributing to the stability and functionality of the enzyme (Fig 3b).

The protein structures of NagS bound to either GlcNAc-6P or 6-PG were virtually indistinguishable from each other with an RMS deviation of only 0.17 for all atoms (Fig 3c). However, the apo-form deviated from the substrate-bound structures. In the substrate-bound conformations, the loops connecting H8 and H9 (residues 226–232) had closed in on the substrate (Figs 3d and S11b), resulting in a maximum main chain shift of 5.8 Å at the Cα of Val229. These loops are located at opposite sides of the dimer, and no other structural shifts were observed, indicating that substrate binding is unlikely to be collaborative. The function of the movable loop is therefore most likely to ensure a compact complex between NagS and GlcNAc-6P, and to trigger catalysis by bringing some of its catalytic side chain residues in contact with the substrate.

### Analysis of the GlcNAc-6P binding site

**Fig 4:**
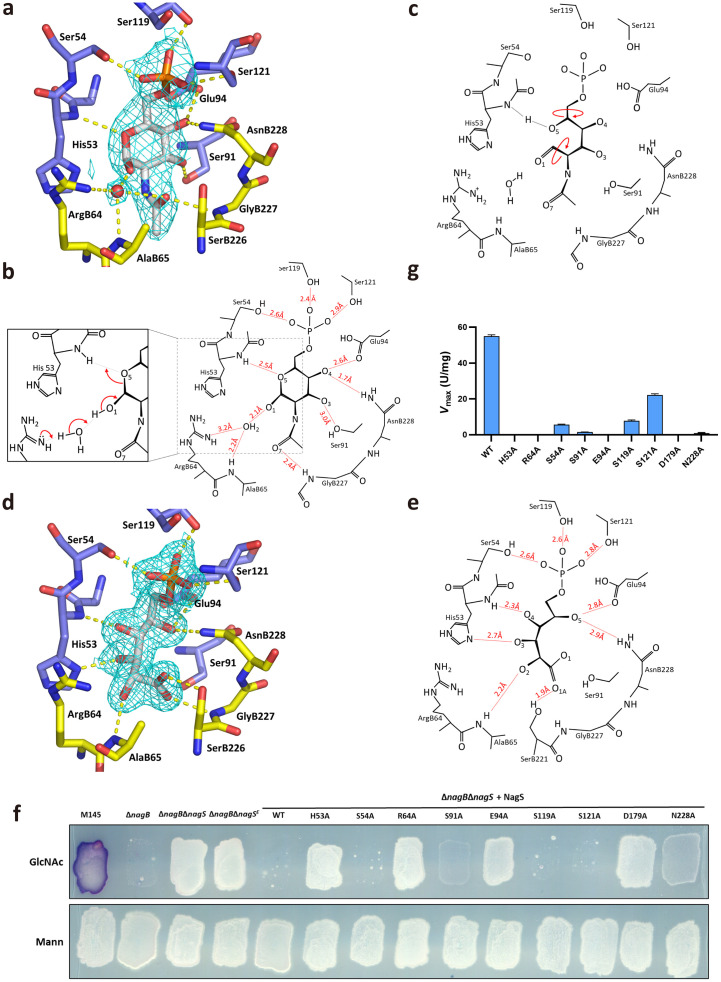
Analysis of the binding site of NagS and activity of NagS mutants. **(a)** NagS active site with bound GlcNAc-6P (gray carbons), protein residues are coloured with pale blue and yellow carbons to indicate the 2 monomers forming the active site. 2|Fo|-|Fc| electron density contoured at 1.2 σ is shown as cyan mesh. Hydrogen bonds are indicated by dashed yellow lines. **(b)** GlcNAc-6P binding site of NagS, with hydrogen bonding distances, or distances between hydrogens and hydrogen bond acceptors indicated. The other molecule of the dimer contributes amino acids ArgB64, AlaB65, GlyB227, and AsnB228. The inset indicates the likely electron rearrangements required for ring-opening, the first step of catalysis. **(c)** Putative transition state after ring-opening, prior to rotations about the C5–C6 and C1–C2 bonds of GlcNAC-6P (indicated in red), that presumably precede subsequent ring closing. These rotations are associated with the rearrangement of hydrogen bonds. This likely requires conformational changes that in the crystal are inhibited by crystal contacts, explaining why the crystals are not enzymatically active. **(d)** NagS active site with bound 6-phosphogluconate (gray carbons), protein residues are coloured with pale blue and yellow carbons to indicate the 2 monomers forming the active site. 2|Fo|-|Fc| electron density contoured at 1.2 σ is shown as cyan mesh. **(e)** Hydrogen bonding distances observed in the 6-phosphogluconic-inhibited state of NagS. The inhibited state suggests how GlcNAc-6P rearranges upon ring-opening, and likely reflects the transition state prior to ring closing, which probably involves Ser91, Glu94, and AsnB228. **(f)** NagS enzyme activity in vivo. GlcNAc sensitivity of ∆*nagB*∆*nagS* harboring clones expressing NagS mutants H53A, S54A, R64A, S91A, E94A, S119A, S121A, D179A, or N228A were grown on MM agar supplemented with 1% mannitol (Mann) and 1% mannitol plus 10 mM GlcNAc (GlcNAc). Single colonies are most likely spontaneous suppressors. **(g)** In vitro activity (*V*_max_) for wild-type NagS (WT) and NagS variants, with the substrate of GlcNAc-6P. The data underlying this Figure can be found in S1 Data.

Clear electron density at the NagS catalytic site demonstrates that residues from both monomers collaborate to form a tight substrate binding pocket (Fig 4a, 4b). Adjacent to this site, a well-ordered water molecule, likely involved in the initial ring-opening step of catalysis, is observed (Fig 4c). Additionally, several other well-ordered water molecules are integral to the active site structure (see S11c Fig). The phosphate groups of GlcNAc-6P and 6-PG engage Ser54, Ser119, and Ser121 from one monomer, resembling the phosphate-binding mechanism seen in MurQ, where three serine residues play a similar crucial role in phosphate-binding [42,43]. The GlcNAc moiety is stabilized through interactions with Ser54, Ser91, and Glu94, as well as the main chain amide of His53 from the same monomer. A putative transition state after ring-opening, prior to rotations about the C5–C6 and C1–C2 bonds of GlcNAC-6P (Fig 4c), may presumably precede subsequent ring closing. Furthermore, GlcNAc-6P interacts with the side chains of ArgB64 and AsnB228, and the main chain amides of AlaB65 and GlyB227 from the adjacent monomer. Similar interactions are found in the 6-PG-bound state (Fig 4d, 4e). We propose that 6-PG is a competitive inhibitor that stabilizes the enzyme in a catalytically relevant state. Its high affinity for NagS is explained by its nine hydrogen bonds with NagS, whereas GlcNAC-6P forms eight direct hydrogen bonds with NagS, and one indirect hydrogen bond through an ordered water molecule.

To investigate the roles of specific residues in binding and catalysis, alanine mutants of all residues that interact with GlcNAc-6P through their side chains together with Asp179, which was determined to be essential for NagS activity from the suppressor mutant, were generated and expressed in the *S. coelicolor* double mutant ∆*nagB*∆*nagS*. The phenotypes of these mutants were analyzed on MM agar plates, with and without GlcNAc. Expression of wild-type *nagS* or its mutant variants, S54A, S119A, or S121A, fully restored GlcNAc sensitivity in the double mutant, indicating that these residues are not crucial for the catalytic activity of NagS. Conversely, mutants S91A and N228A only partially restored GlcNAc sensitivity, underscoring their relevance in the enzyme’s catalytic function. However, constructs expressing the H53A, R64A, E94A, or D179A mutants of NagS showed no restoration of GlcNAc sensitivity, indicating that these residues are essential for NagS activity (Fig 4f).

To corroborate these findings, we determined the kinetic parameters of all of the NagS variants in vitro, which aligned closely with our in vivo observations (Table 1 and S12 Fig). Variants S54A, S119A, and S121A displayed relatively small changes in *K*_m_ and *k*_cat_ values, compared to the other mutants, confirming these residues are not crucial for catalysis. In contrast, variants S91A and N228A exhibited significantly reduced activity, with very low *V*_max_ values (Fig 4g). Specifically, S91A demonstrated a 40-fold decrease in *k*_cat_/*K*_m_, while N228A showed a 208-fold decrease in *k*_cat_/*K*_m_ and a 3.9-fold reduction in *K*_m_, indicating that they are important for catalysis. Furthermore, the variants H53A, R64A, E94A, and D179A were completely inactive (Table 1 and Fig 4g), confirming that these residues are critical for catalysis.

### A novel promiscuous function for NagA as 6P-chromogen I deacetylase

**Fig 5:**
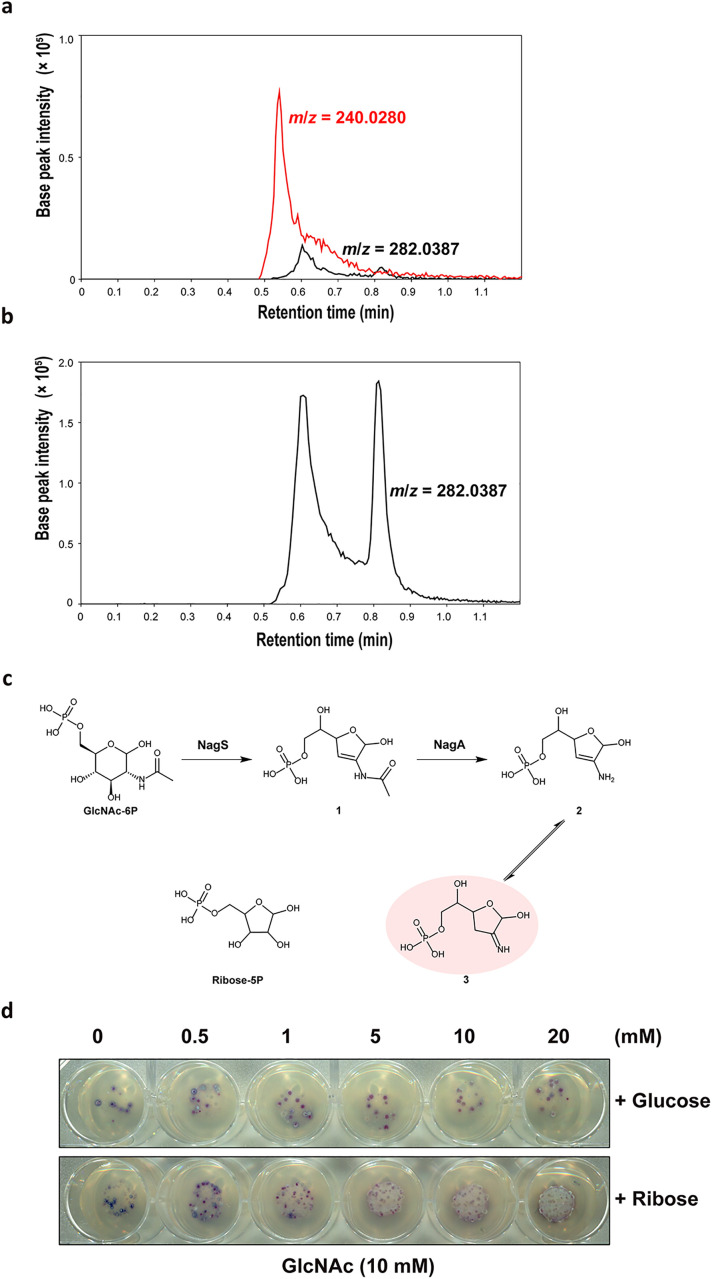
New function of *S. coelicolor* NagA and an updated amino sugar metabolic pathway. Extracted ion chromatograms for compound **1** (*m*/*z* 282.0387, shown in black lines) and compound **2**/**3** (*m*/*z* 240.0280, shown in red lines) in the enzymatic reaction mixture of GlcNAc-6P with NagS and NagA (**a**) or deactivated NagA **(b)**. **(c)** Updated metabolic pathway of amino sugar in *Streptomyces*. Based on the metabolic pathway in S1 Fig, we propose a new metabolic route in GlcNAc metabolism. Apart from the canonical reaction whereby GlcNAc-6P is metabolized by NagA and NagB to fructose-6P, GlcNAc-6P can also be dehydrated by NagS and subsequently deacetylated by NagA to form compound **3** (shown in light red), whose is a likely toxic compound whose chemical structure is similar to ribose-5P. **(d)** Ribose by-passes GlcNAc toxicity. Spores (5 × 10^5^ CFU) of the *S. coelicolor* M145 *nagB* mutant were spotted on MM supplemented with 1% mannitol plus 10 mM GlcNAc and different concentrations (0–20 mM) of either glucose or D-ribose, followed by incubation for 72 h at 30 °C. Note that 1 mM ribose or more alleviates GlcNAc toxicity and allows the cells to grow, while even at 20 mM glucose the colonies still are sensitive to GlcNAc. Single colonies are most likely suppressors.

The observation that the deletion of *nagA* relieves the toxicity of GlcNAc to *nagB* null mutants created a mystery in terms of the enzyme’s role in GlcNAc metabolism. NagS is the first step towards a toxicity pathway, and in the absence of NagA, the NagS substrate GlcNAc-6P is expected to accumulate in large amounts when cells grow on GlcNAc, which should therefore be more, rather than less, toxic to the cells. However, *nagA nagB* double mutants grow very well on GlcNAc. The most likely explanation was that NagA has an additional role besides deacetylating GlcNAc-6P. We therefore wondered if NagA might also deacetylate 6P-chromogen I (**1**), the reaction product of NagS. To test this, *S. coelicolor* NagA was expressed in *E. coli* and purified to homogeneity, and its activity with different substrates was tested. NagA deacetylated GlcNAc-6P, ManNAc-6P and GalNAc-6P in vitro, with a clear preference for GlcNAc-6P (*k*_cat_/*K*_m_ of 1.01 × 10^5^ M^-1^·s^−1^) as compared to 6.06 × 10^3^ M^−1^·s^−1^ for ManNAc-6P and 1.03 × 10^4^ M^−1^·s^−1^ for GalNAc-6P (Table 1 and S13a–S13c Fig). These kinetic parameters are comparable to those for NagA isozymes reported in *E. coli* and *Mycobacterium tuberculosis* [44,45], which shows that the purified NagA was fully active in vitro. Next, we tested if NagA could act on **1**. GlcNAc-6P was first incubated with NagS at 30 °C; next, either active NagA or heat-inactivated NagA was added, and the reaction mixture was analyzed by LC–MS. No difference in peak intensity was seen when heat-inactivated NagA was added, showing that **1** was stable under the given reaction conditions. Excitingly, **1** disappeared after adding active NagA, and new products with an exact mass of 240.0280 (S6 Table) were detected in the reaction mixture (Fig 5a, 5b). This shows that NagA is a promiscuous enzyme, whereby besides its textbook function it can also deacetylate **1**, the product of NagS. The HRESIMS spectrum of the peaks established a molecular formula of C_6_H_12_NO_7_P (*m/z* 240.0280), which is the deacetylated product of **1** (S13d Fig).

Thus, our work reveals a novel metabolic route in central amino sugar metabolism (Fig 5c). Herein, GlcNAc-6P is dehydrated by NagS to **1**. Subsequently, **1** is deacetylated by NagA to form enamine **2** (2-amino-2,3-dideoxy-6-phosphate-D-erythro-hex-2-enofuranose), which is unstable and spontaneously converts to the corresponding imine **3** (2-imino-2,3-dideoxy-6-phosphate-D-erythro-hexofuranose).

### Supplementation of ribose relieves GlcNAc toxicity to *nagB* mutants

We then wondered what the basis might be for the toxicity of compound **3**. We noticed that imine **3** shows significant structural similarities to ribose-5-phosphate, an intermediate in pentose metabolism and the sugar moiety of nucleic acids. This suggests that **3** may interfere with the synthesis of nucleotides when accumulating at high concentrations. If this is the case, high concentrations of ribose may relieve the toxicity. Therefore, either ribose or glucose was added to cultures of the *S. coelicolor nagB* mutant grown in the presence of GlcNAc, to determine if these sugars could alleviate the toxicity of GlcNAc. Importantly, *nagB* mutants were significantly less sensitive to GlcNAc when grown in the presence of higher concentrations of ribose, while glucose did not alter the sensitivity (Fig 5d). This provided an important clue as to the toxicity of **3**, which may act by interfering with ribose metabolism, and hence with the synthesis of nucleotides.

## Discussion

**Fig 6:**
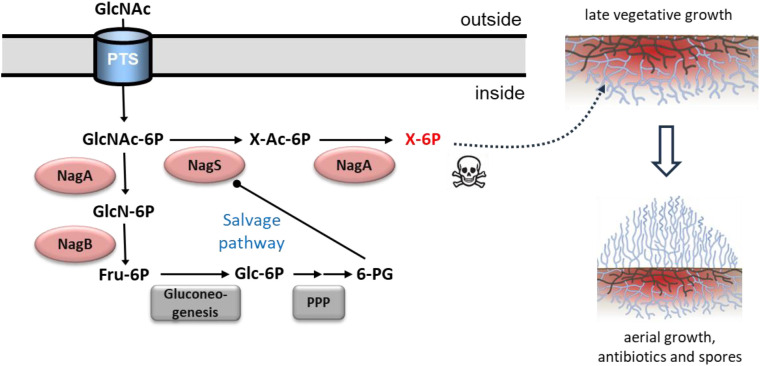
Model for the metabolic control of development by GlcNAc and NagS. During late vegetative growth of streptomycetes, the old vegetative or substrate hyphae are degraded in a process of programmed cell death (PCD), to produce the nutrients required to build the aerial mycelium (see mycelial drawings on the right). Mycelial lysis results in breakdown of the cell-wall, leading to the accumulation of GlcNAc-6P, which is a major nutritional signal for the onset of development and antibiotic production [18]. NagS converts GlcNAc-6P into 6P-chromogen I (denoted as X-Ac-6P), which in turn is deacetylated by NagA into a toxic metabolite (denoted as X-6P) that resembles ribose (Fig 5). The toxic metabolite promotes cell lysis, thus releasing more GlcNAc-6P that serves as substrate for NagS and NagA. A *salvage pathway* then switches off the toxic pathway again. For this, GlcNAc-6P is converted by NagA and NagB into Fructose-6P (Fru-6P), which enters the pentose phosphate pathway (PPP), thereby producing 6-phosphogluconate (6-PG), a metabolic inhibitor of NagS (see Fig 4). Thus, production of toxic metabolites ceases and the transition to aerial growth can be initiated. Arrows with round ends represent inhibition, dashed arrow shows proposed activity.

*N*-acetylglucosamine (GlcNAc) is a preferred carbon source for streptomycetes and stands at the crossroads of amino sugar metabolism, glycolysis and cell-wall synthesis. The molecule plays a key role in nutrient sensing and in the ultimate decision to initiate sporulation and antibiotic production [18]. Our work shows that the new enzyme *N*-acetylglucosamine-6P dehydratase is a central player in GlcNAc sensing in *Streptomyces* and is the gateway to a previously undiscovered toxicity pathway (see below and model in Fig 6). GlcNAc sensing is lost in *nagS* mutants on both MM and R5 agar, with *nagS* mutants being unaffected by GlcNAc in terms of development and the production of secondary metabolites such as Act, Red, and siderophores. NagS is highly conserved across – and limited to – *Streptomycetaceae*, namely *Streptomyces*, *Kitasatospora*, and *Streptacidiphilus* (Fig 2). The lack of NagS orthologues outside the *Streptomycetaceae* may be explained by the life cycle-dependent role for NagS. Interestingly, gene synteny analysis shows very strong evolutionary linkage between *nagS* and *dmdR1*, which encodes the iron master regulator DmdR1. This relates NagS to iron homeostasis, and indeed, our data show that repression of siderophore production by GlcNAc is lost in *nagS* null mutants (S2 Fig). In the context of our work, it is important to note that Fe^2+^ contributes to cell death prior to development [21]. And the relationship between iron accumulation, NagS and the onset of development deserves more attention.

GlcNAc-6P dehydratase NagS converts GlcNAc-6P to **1**, which is subsequently deacetylated via a previously unknown promiscuous enzymatic activity of NagA to enamine **2**, which spontaneously converts to the corresponding imine **3** (Fig 5c). Our findings provide a basis for incorporation into genome-scale metabolic models of *S. coelicolor* and other streptomycetes, which may improve their predictive accuracy and place the proposed pathway within the context of the organism’s overall metabolism, potentially revealing so far unrecognized interactions. The reaction catalyzed by NagS most likely starts with a dehydration step that involves opening the glucose 6-ring, followed by its closure into a 2′,3′-dideoxy-2′,3′-unsaturated ribose 5-ring. This transformation likely begins with the ring-opening of the glucose moiety in GlcNAc-6P, and we propose this process mirrors the ring-opening mechanism of glucose-6-phosphate by phosphoglucose isomerase [46]. ArgB64 in NagS likely assumes a role similar to Lys518 in phosphoglucose isomerase through a coordinating a water molecule (Fig 4c). The acidic Asp179 then forms a salt bridge with ArgB64. Its mutation to Asparagine or Alanine would prevent the guanidinium group of ArgB64 from properly orienting to attack O1 of the GlcNAc-6P substrate. This ring-opening likely precipitates a series of rotations around single bonds within the carbohydrate, aligning it into a configuration similar to that of the linear inhibitor 6-PG. However, 6-PG is distally stabilized through an interaction between its O1A and the side chain of SerB221, an interaction unlikely to occur with the linearized GlcNAc-6P due to steric hindrance caused by its longer carbohydrate chain. We hypothesize that subsequent bond formation between O1 and C4, coupled with dehydrolysis removing O3 and forming a double bond between C2 and C3, is facilitated by Glu94, with the support of Ser91 and AsnB228. Although our data do not conclusively detail these latter steps, the successful capture of GlcNAc-6P-bound to the active site in pre-formed crystals indicates that substrate reorientation—necessitating rotations around single bonds that precede ring closing and dehydrolysis—likely requires conformational rearrangements of the protein, which were impeded by crystal contacts.

### Metabolic toxicity and nutrient signaling in *Streptomyces*

Lysis of the vegetative or substrate mycelia is a key event during colony growth and development, through a process of PCD. Cytotoxic compounds with antiproliferative activity such as prodiginines [17] or anthracyclines [47] play a major role in the onset of PCD in streptomycetes, by killing biomass in the center of the colony [17]. The accumulation of GlcNAc around the colonies is perceived by *Streptomyces* as the start of lytic degradation of the cell-wall, a major signal for the onset of development. Our data show that amino sugar toxicity mediated by NagS and NagA is required for GlcNAc sensing during the control of early development. Indeed, in the absence of *nagS* or *nagA*, GlcNAc sensing is lost. Deletion of *nagS* in the wild-type strain did not affect the timing of aerial development, but led to reduced formation of aerial biomass. This will negatively affect sporulation, which is a major competitive disadvantage in the natural habitat. The fact that compound **3** structurally resembles ribose-5P provided a clue as to the nature of the toxicity. Indeed, the addition of ribose relieved the toxicity of GlcNAc to *nagB* mutants, which suggests that the toxicity pathway governed by NagS and NagA might interfere with nucleic acid synthesis. This raises the interesting possibility that compound **3** could modulate enzymes utilizing ribose-5P, which warrants further investigation in future studies.

Based on our data, we propose a model for the role of NagS in GlcNAc signaling (Fig 6). As vegetative growth progresses, degradation of the substrate mycelium is initiated to provide the required for the buildup of the new aerial biomass. Cell-wall lysis results in the release of GlcNAc, which serves as a substrate for the toxicity pathway governed by NagS and NagA. The accumulation of the toxic compound will aid in promoting cell death. In turn, the activity of NagS is metabolically inhibited by 6-PG. 6-PG is an intermediate of the pentose phosphate pathway (PPP) and we therefore propose a *salvage pathway* (Fig 6). 6-PG is derived in just a few metabolic steps from GlcN-6P, by its conversion to fructose-6P by NagB, then to glucose-6P by phosphoglucose isomerase (Pgi), and to 6-PG via the PPP. This probably also explains why *nagB* mutants are hyper-sensitive to GlcN-6P or GlcNAc-6P; after all, *nagB* mutants cannot convert GlcN-6P into Fru-6P and then to 6-PG, thus blocking the salvage pathway, so that NagS stays active. GlcNAc-6P is a key nutrient for streptomycetes, and once levels are low again, aerial growth is initiated. In laboratory experiments using agar plates the pool of GlcNAc (some 20 ml of agar containing 10 mM GlcNAc) can never be depleted by the colonies, so that development is permanently blocked. Therefore, the discovery of NagS appears to be the final piece of the jigsaw in terms of GlcNAc signaling and the control of development.

In conclusion, we report on the function and structure of the amino sugar dehydratase NagS, a novel enzyme that forms a gateway for GlcNAc sensing in *Streptomyces*. We thereby provide new insights into the molecular basis for the link between amino sugar metabolism and the control of development and antibiotic production in streptomycetes.

## Materials and methods

### Bacterial strains, culture conditions, plasmids, and oligonucleotides

All strains described in this work are listed in S1 Table. *Escherichia coli* was grown and transformed according to standard procedures [48], with *E. coli* JM109 serving as the host for routine cloning, and *E. coli* ET12567 [49] for the isolation of nonmethylated DNA for transformation into *Streptomyces* [50]. For protein heterologous expression, *E. coli* Rosetta (DE3)pLysS was used for NagS and *E. coli* BL21 (DE3) was used for NagA. *E. coli* was grown in Luria-Bertani (LB) media in the presence of selective antibiotics as required.

*Streptomyces coelicolor* A (3)2 M145 [51] served as the parent of all mutants. *S. coelicolor nagB* mutants, ∆*nagB*, and *nagAB* mutants, ∆*nagAB*, and *nagB* suppressor mutants SMA11, have been described previously [30]. All *Streptomyces* media and routine techniques are described in the *Streptomyces* manual [50]. SFM (soy flour mannitol) agar was used for conjugation and cultivation of the spores. Phenotypic characterization was done on R5 medium and MM with 1% (w/v) mannitol supplemented with sugars as stated and, where appropriate, with the antibiotics apramycin (20 μg/ml) and/or thiostrepton (20 μg/ml) as selective markers.

All plasmids and oligonucleotides are summarized in S1 and S2 Tables of the supplemental material, respectively. The shuttle vector pSET152 [52] was used for genetic complementation and over-expression experiments, while the unstable multi-copy shuttle vector pWHM3 [53] was exploited for gene replacement strategies [54]. Cre recombinase expressing plasmid, pUWLcre [55] was used for the creation of deletion mutants via genetic excision via *loxP* marked sites (see below for details). Expression vector pET-15b and pET-28a (+) (Novagen) were used for proteins heterologous expression. All DNA sequencing was performed by BaseClear BV (Leiden, the Netherlands).

### Gene knock-out, complementation and over-expression

The detailed procedure for the creation of *S. coelicolor* gene replacement and deletion mutants is described previously [56]. Gene replacement mutants were generated via homologous recombination, with the gene of interest replaced by the apramycin resistance cassette *aac(C)IV*. For this, the upstream and downstream flanking regions of *nagS* were PCR-amplified from *S. coelicolor* M145 genomic DNA using primer pairs SCO4393-LF/LR and SCO4393-RF/RR and cloned into pWHM3 using engineered *Eco*RI/*Xba*I and *Xba*I/*Hind*III restriction sites, respectively. The apramycin resistance cassette flanked by *loxP* sites was cloned in-between as an *Xba*I fragment. The resulting knock-out plasmid, designated pKO-4393, was introduced into *S. coelicolor* and ∆*nagB* via protoplast transformation. Correct recombination events were verified by appropriate antibiotics resistance and confirmed by PCR. To obtain deletion mutants, the apramycin resistance cassette was excised by introduction of the Cre recombinase expressing plasmid, pUWLcre [55], which allows for efficient removal of the cassette via the *loxP* recognition sites [57]. Deletion mutants were checked for the appropriate antibiotic sensitivity (loss of apramycin resistance) and confirmed by PCR.

For the complementation of *nagS*, DNA fragment of *nagS* coding region was amplified from the *S. coelicolor* genome DNA and cloned into pSET152, giving the vector pCOM-4393. NagS site-directed mutated sequences were amplified using two pairs of primers: 4393-compF/4393-compR and the corresponding mutated primers. The two amplified fragments were cloned into pSET152 by Gibson assembly [58], producing mutated *nagS* complementary vectors. The complementary constructs were introduced into *nagB*-*nagS* double mutant to obtain the corresponding complemented strain. For *nagS* and *nagA* over-expression, a 796-bp DNA fragment containing *nagS* coding region and a 1185-bp DNA fragment containing *nagA* coding region were amplified with the primers SCO4393-OE-F/R and SCO4284-OE-F/SCO4284-OE-R, and ligated simultaneously with the 310-bp highly efficient promoter *ermE* [59] into pSET152 to produce *nagS*-overexpressing vector pOE-4393 and *nagA*-overexpressing vector pOE-4284. The vectors were transformed into Δ*nagB* to generate *nagS* and *nagA*-overexpressing strains, respectively.

### Heterologous expression and purification of *S. coelicolor* NagA

For heterologous expression of *S. coelicolor* NagA in *E. coli*, the 1146-bp *nagA* coding regions was amplified from genome DNA using primer pair NagA-exp-F/NagA-exp-R. The PCR fragment was ligated into pET-28a (+) from *Xho*I and *Nco*I, generating expression vector pEX-4284. The NagA expression vector was transformed into *E. coli* BL21(DE3), and the expression of C-terminal His_6_-tagged NagA recombinant protein was induced by addition of Isopropyl β-D-1-thiogalactopyranoside at a final concentration of 1.0 mM when the cell density reached around an optical density at 600 nm of 0.6. In addition, 1 mM ZnCl_2_ was also supplemented into the cells as a range of NagA isozymes require divalent metal cations for their function [60,61], followed by incubation at 26 °C overnight. Cells were harvested, washed, and disrupted in lysis buffer (50 mM sodium phosphate, 300 mM NaCl, 10 mM imidazole, pH 7.4) by sonication on ice. The preparations were then centrifuged, and the soluble His_6_-tagged NagA from supernatant was purified using HisPur Cobalt Resin (Thermo Fisher Scientific; USA). After properly washing with wash buffer (50 mM sodium phosphate, 300 mM NaCl, 10 mM imidazole, pH 7.4), recombinant NagA was eluted from the resin with elution buffer (50 mM sodium phosphate, 300 mM NaCl, 300 mM imidazole, pH 7.4), and then desalted into phosphate buffer (25 mM phosphate pH 7.4, 100 mM NaCl, 1 mM DTT) and stored at −80 °C.

### Phylogenetic analysis of NagS

Protein homology searches were performed using BLASTp [62]. The phylogenetic tree of different SIS containing proteins was made by MEGA11 [63]. SyntTax was used for gene synteny [64]. Protein alignments were analyzed by Clustal Omega (www.ebi.ac.uk/Tools/msa/clustalo/) and visualized using Jalview (Version 2.11.2.7).

For comprehensive NagS phylogenetic analysis, *S. coelicolor* NagS is used as a query to search StringDB (v12.0 10.1093/nar/gkac1000) proteins using diamond (version 2.1.8, 10.1038/s41592-021–01101-x) with “--sensitive” switch and a default *e*-value threshold of 0.001 (S4 Table). Proteins found are extracted from the database and aligned using MAFFT (version 7.520, 10.1093/molbev/mst010) with a maximum of 1,000 iterative refinement. The alignment is then piped into FastTree (version 2.1.11, [65]) to make a tree with bootstrap values of 1,000 repeats. The phylogenetic tree produced by FastTree is then uploaded to iTOL (version 7.0, 10.1093/nar/gkae268) to produce the figure. The corresponding tree file and annotation dataset can be found in Supplementary material.

### Heterologous expression and purification of NagS

For in vitro enzyme experiments and structure elucidation via X-ray crystallography, N-terminally His_6_-tagged NagS, its variants with site-directed mutations (H53A, S54A, R64A, S91A, E94A, S119A, S121A, D179A, and N228A) were expressed in *E. coli* Rosetta (DE3)pLysS (Novagen). Briefly, the *nagS* coding sequence was PCR-amplified from *S. coelicolor* genomic DNA or synthesized DNA fragments, while its site-directed mutated sequences were amplified from the vectors for mutated NagS complementation (pCOM-4393-H53A to pCOM-4393-N228A). The amplified fragments were cloned into pET-15b to obtain the expressing plasmids, which were transformed into *E. coli* Rosetta (DE3)pLysS competent cells. Expression host cells were grown at 37 °C with shaking at 200 rpm in LB media supplemented with chloramphenicol (25 μg/ml) and ampicillin (100 μg/ml) until an OD_600_ of 0.6–0.8 was reached. Protein expression was induced with isopropyl β-D-1-thiogalactopyranoside to a final concentration of 0.5 mM, followed by further incubation at 37 °C with shaking at 200 rpm for 4 h. His_6_-tagged NagS and its variants were purified using a Ni-NTA column (GE Healthcare) with Isolation Buffer (500 mM NaCl, 5% glycerol, 50 mM HEPES, 10 mM β-mercaptoethanol, pH 8.0) containing 250 mM imidazole, as described [66]. Fractions containing the target proteins were pooled and concentrated before further purification by size-exclusion chromatography (Superdex 200) on an AKTA Pure FPLC system (Cytiva) with Isolation Buffer. Proteins were desalted with HEPES buffer (20 mM HEPES, 300 mM NaCl, 5% glycerol, 1 mM DTT, pH 7.4) prior to use in crystallization trials and enzyme assays.

### Enzyme assays

All enzyme experiments were performed at 30 °C using a Cary 60 UV-Vis spectrophotometer (Agilent). Each kinetic assay was carried out in triplicate and in quartz cuvettes (Hellma) with a path length of 10 mm. The initial velocity (*V*_0_) measurements were reproducible within 10% error. All stocks of enzymes and substrates were kept on ice during the entire experiments. The chemicals used were purchased from Sigma–Aldrich unless stated otherwise.

For NagS kinetic analysis, the rate of reaction was measured by following the increase in UV absorbance at 230 nm as a function of time. The reaction (300 μl in total) was started by the addition of the substrate (final concentration 0–5 mM) to a mixture containing 150 μl phosphate buffer (100 mM phosphate, 100 mM NaCl, pH 7.4) and 220 nM NagS. The extinction coefficient of the product of NagS was experimentally determined to be 2.17 × 10^3^ M^−1^·cm^−1^ (S14 Fig).

Kinetic analysis of *S. coelicolor* NagA was performed using a previously described direct continuous spectrophotometric assay [44]. Here, the rate of reaction was obtained from measurement of the decrease of the amide absorbance of GlcNAc-6P/ManNAc-6P/GalNAc-6P at 215 nm. The reaction mixture (300 μl) contained 150 μl phosphate buffer, *S. coelicolor* NagA (150 nM for GlcNAc-6P and 400 nM for ManNAc-6P and GalNAc-6P) and the substrates, and the reaction was initiated by the addition of substrate (concentration from 0.33 to 6.67 mM). The extinction coefficients were 500 M^−1^·cm^−1^ for GlcNAc-6P, 408 M^−1^·cm^−1^ for ManNAc-6P and 612 M^−1^·cm^−1^ for GalNAc-6P, all determined spectrophotometrically. For all kinetics, initial rate data were analyzed and fitted with the Michaelis-Menten model using GraphPad Prism (version 8.3.0).

### Identification of the products of the enzymatic reactions

NagS product was identified by a combination of Nuclear Magnetic Resonance (NMR) and LC–MS analysis. The reaction samples for NMR were prepared in phosphate buffer, in which 6.67 mM GlcNAc-6P/ManNAc-6P was incubated with 220 nM NagS at 30 °C, and a sample with boiled NagS was used as a control. After reaching equilibrium at 10 min, the reaction mixture was freeze-dried and then dissolved in 170 μl D_2_O, and transferred to NMR tubes. The NMR spectra were acquired on a Bruker AVIII-600 spectrometer (Bruker BioSpin GmbH) at a field strength of 600 MHz.

### Structural data collection and structure determination

Purified NagS at a concentration of 15–20 mg/ml was screened for crystallization by sitting-drop vapor-diffusion using the PGA Screen (Molecular Dimensions), Clear Strategy Screens CSS-I and CSS-II (Molecular Dimensions), JCSG+ (Qiagen/Molecular Dimensions), and the PACT screen (Molecular Dimensions) as well as optimization screens at 20 °C. The 75 μl reservoir of 96-well Innovaplate SD-2 plates was pipetted by a Genesis RS200 robot (Tecan) and drops were made by an Oryx6 robot (Douglas Instruments). Hexagonal NagS crystals (space group P6_5_22) were obtained from JCSG number 83 (96-well G11), which consisted of 2.0 M Ammonium sulphate, 0.1 M BIS-Tris, pH 5.5. Crystals were soaked in mother liquor with 10%–20% glycerol as cryoprotectant, that included no other additives, or either 100 mM of the substrate GlcNAc-6P, or 200 mM of the inhibitor 6-phosphogluconate. After loop mounting, they were flash-frozen in liquid nitrogen.

X-ray data were collected at the European Synchrotron Radiation Facility (Grenoble, France) on beamline ID-23 for the apo-enzyme: 1,410 frames were collected on a Pilatus 6M detector at an X-ray wavelength of 0.9724 Angstroms, an exposure time of 0.037 s, transmission of 10% and an oscillation range of 0.2°. NagS in complex with GlcNAc-6P data were collected on beamline ID-29 with a Pilatus 6M detector. For the native crystal, 1,020 images were collected at 1.2727 Å wavelength with an exposure time of 0.02 s, transmission of 100% and an oscillation range of 0.05°. We collected 680 frames at 0.976251 Angstroms wavelength with an exposure time of 0.02 s, transmission of 47.34% and an oscillation range of 0.1°. XDS [67] was used to process all the data collected. Scaling and merging were done using the CCP4 program ‘aimless’ [68]. Subsequent structural analysis of the crystals soaked in substrate, revealed that the crystals did not significantly catalyze its conversion, see Results section. The diffraction data of the 6-PG inhibited crystals were collected at the Diamond synchrotron radiation facility on beamline I04. 3,600 images were collected at a wavelength of 0.9 Å with an exposure of 0.05 s, transmission of 100% and oscillation of 0.05°. Data processing was performed via Xia2 [69]. The resolution of the inhibited crystal form was 1.7 Å, which was significantly higher than the apo- and substrate-bound crystals (2.3 Å and 2.6 Å, respectively).

The structure of apo-NagS was solved by molecular replacement using the structure of a putative phosphoheptose isomerase from *B. halodurans* C-125 (PDB code 3CVJ) as the model. Two subunits were present in the asymmetric unit, and in the initial stages of structure refinement, noncrystallographic symmetry restraints were imposed. Clear densities corresponding to well-ordered water molecules and either 6-PG, or GlcNAc-6P for the substrate-bound crystal forms, were observed for all three crystal forms. Models that included the inhibitor, or the substrate where appropriate, were inspected and interactively rebuilt using Coot [70,71].

Water molecules were introduced automatically in refinement using Phenix [72]. Then, noncrystallographic symmetry restraints were removed and six consistent TLS fragments per monomer were automatically generated for the inhibited crystal form and refined by Phenix. Refinement of the apo- and substrate-bound structures benefitted from reference model torsion angle restraints provided by the inhibited structure, as judged by a significant improvement in R-free. TLS refinement using the same fragments as in the inhibited structure, improved the substrate-bound, but not the apo-structure. S3 Table shows the data collection and refinement statistics for the data sets obtained.

### ITC assays

To identify the possible substrates of NagS, ITC tests were performed using a MicroCal PEAQ-ITC Automated microcalorimeter (Malvern Panalytical Ltd, Malvern, UK). A 700 µM solution of substrates (GlcNAc-6P, ManNAc-6P, or GalNAc-6P) in 20 mM Tris pH 7.4, 100 mM NaCl, 50 µM DTT, was titrated into a 50 µM enzyme preparation in the same buffer. Control titrations included the titration of buffer into enzyme and substrate into buffer. The samples were equilibrated to 25 °C prior to measurement. The titrations were conducted at 25 °C under constant stirring at 750 rpm. Each experiment consisted of an initial injection of 0.4 µL followed by 18 separate injections of 2.0 µL into the sample cell of 200 µL. The time delay between each injection was 180 s, the measurements were performed with the reference power set at 5 μcal∙s^−1^ and the feedback mode set on “high”. The calorimetric data obtained were analyzed using MicroCal PEAQ-ITC Analysis Software Version 1.20 (Malvern Panalytical Ltd, Malvern, UK). ITC data fitting was made based on the software’s “one set of sites” fitting model. The best fit is defined by chi-squared minimization. Thermodynamic parameters are reported as the average of three experiments with the standard deviation, unless stated otherwise.

### Analytical size-exclusion chromatography

The oligomeric state of purified NagS was characterized by analytical size-exclusion chromatography. Protein at various concentrations (35 μM and 70 μM) was injected onto a Superdex 200 10/300 GL gel-filtration chromatography column (Cytiva) equilibrated in a buffer containing 20 mM HEPES pH 7.5, 300 mM NaCl, 5% glycerol, and 1 mM DTT. A calibration curve (S5 Fig) relating elution volume (S7 Table) to molecular weight was generated by injecting a Protein Standard Mix (15–600 kDa; Sigma, product number: 69385) onto the same column with the same buffer.

### Extinction coefficient measurement of the product of NagS

To measure and calculate the extinction coefficient of NagS, an indirect method was used instead of spectrophotometry. As only a portion of the substrate is converted by NagS after the reaction, the concentration of the product is obtained by subtracting the final concentration of GlcNAc-6P, from the initial concentration of GlcNAc-6P (3.33 mM). First, the standard curve of the linear relationship between the concentrations of GlcNAc-6P (1–5 mM) and the peak areas of GlcNAc-6P measured by LC-MS was constructed (S14 Fig). The peak areas of GlcNAc-6P after the reaction were also measured (mean = 31467.63), thus the concentration of the remaining GlcNAc-6P was calculated as 2.15 mM using the standard equation. Thus, the concentration of NagS product was calculated as 1.18 mM (initial substrate concentration 3.33 mM minus final substrate concentration 2.15 mM). The final absorbance caused by the product was measured spectrophotometrically, which was 2.564. Therefore, the extinction coefficient of the product of NagS was calculated as 2.17 × 10^3^ M^−1^ cm·^1^ by dividing the absorbance brought by the product by its concentration. All measurements involved were done in triplicate.

### NagS-NagA coupled enzymatic assays

For NagS-NagA coupled assay, the reactions were carried out in the volatile buffer (50 mM *N*-ethylmorpholine/acetate pH 7.4), which is compatible with the subsequent LC–MS analysis. GlcNAc-6P (10 μl, 100 mM) was first incubated with NagS, followed by the addition of 150 nM NagA or heated-inactivated NagA after the NagS-catalyzed reaction reached equilibrium. The reaction mixture was monitored at 230 nm until the absorbance stabilized, and then subjected to mass spectrometry analysis.

### Thermal denaturation assays

The thermal stability and melting point of the protein were identified through measuring the intensity of SYPRO orange fluorescence. Upon the unfolding of the protein when temperature rises, the intensity of SYPRO orange increases due to the increased exposure of hydrophobic regions [73]. This change is monitored using a TOptical Real-Time qPCR Thermal Cycler (Biometra). NagS was tested for the thermal ability with or without 6-PG. For this, 200 μl of NagS at 4 μM was made up using the isolation HEPES buffer and 5X SYPRO orange (Invitrogen). A set of 3 repeats with a final volume of 50 μl of the protein solution per well was dispensed into a MicroAmp Optical 96-Well Reaction Plate (Applied Biosystems) and sealed by a Microseal ‘B’ seal (BIO-RAD). The plate was then centrifuged at 2,000 rpm for 2 min at 10 °C using an Allegra X-15R centrifuge fitted with a µSX4250 rotor prior to the experiment. The level of SYPRO orange intensity was monitored between 20 and 75 °C. Additionally, 6-PG (2, 5, and 10 mM) was tested to determine its possible effect on the thermal stability of NagS.

### Inhibitor kinetic assays

The inhibitory activity of 6-PG with respect to the enzymatic activity of NagS was determined. For this, the rate of reaction was measured by following the increase in UV absorbance at 230 nm as a function of time. Each assay (300 μl in total) was initiated by the addition of GlcNAc-6P (final concentration 0–8 mM) to a mixture containing 150 μl phosphate buffer (100 mM phosphate, 100 mM NaCl, pH 7.4), 6-PG (0.33 or 1 mM), and 220 nM NagS. Initial rates were analyzed and fitted with the competitive inhibition model using GraphPad Prism (version 8.3.0). The rate equation used for *K*_i_ calculation is *K*_m_’ = *K*_m_*(1+[*I*]/*K*_i_).

### Detection of compound 1–3 by LC–MS

For LC–MS analysis, the reactions were performed in a volatile buffer. In brief, 1 mM GlcNAc-6P was incubated in 50 mM *N*-ethylmorpholine acetate pH 7.4 at 30 °C, and the reactions were initiated by the addition of 220 nM active NagS or boiled NagS (for the control sample). After the absorbance of the reaction mixtures had stabilized at 230 nm, the enzyme in the reaction mixture was precipitated with methanol and the supernatant obtained was then used for LC–MS analysis. LC–MS/MS acquisition was performed using a Shimadzu Nexera X2 UHPLC system, with attached PDA, coupled to a Shimadzu 9030 QTOF mass spectrometer, equipped with a standard ESI source unit. A total of 2 µL of each sample was injected into a Waters Acquity HSS C_18_ column (2.1 × 100 mm), which was run at a flow rate of 0.5 ml min^−1^ using 0.1% formic acid in H_2_O as solvent A, and 0.1% formic acid in acetonitrile as solvent B. The elution gradient used was 5% B for 1 min, 5%–17% for 1 min, 17%–20% for 8 min, then 20%–100% for 1 min. All samples were analyzed in negative polarity, using data dependent acquisition mode, in which full scan MS spectra (*m/z* 100–1,700, scan rate 10 Hz, ID enabled) were followed by two data dependent MS/MS spectra (*m/z* 100–1,700, scan rate 10 Hz, ID disabled) for the two most intense ions per scan at a collision energy of 20 eV. The parameters used for the ESI source were: interface voltage −3 kV, interface temperature 300 °C, nebulizing gas flow 3 L min^−1^, and drying gas flow 10 L min^−1^.

## Supporting information

S1 Fig Metabolic pathway of aminosugar in *Streptomyces.* Peptidoglycan degradation releases monomers of GlcNAc and MurNAc, which are subsequently taken up by the cells for recycling. The phosphoenolpyruvate-dependent phosphotransferase system (PTS) phosphorylates monomeric GlcNAc during transport into GlcNAc-6P. Subsequently, GlcNAc-6P is metabolized by NagA and NagB to Fru-6P, which enters into glycolysis. Limited information is available on GlcN transport and metabolism in *S. coelicolor*. Besides its metabolism to fructose-6P, GlcN-6P is also the starting point for the biosynthesis of Lipid II, the building block for cell-wall synthesis. GlcNAc-6P and GlcN-6P are effector molecules for DasR, which is a global repressor of among others aminosugar metabolism, natural product biosynthesis, and development in *Streptomyces*. Metabolic routes are represented by arrows with corresponding enzymes. For clarity, the substrates and enzyme names are abbreviated. Abbreviations not mentioned in the text: Glk, glucokinase; Pgi, glucose-6-phosphate isomerase; DasD, *N*-acetyl-β-D-glucosaminidase.
(TIF)

S2 Fig Inhibition of siderophore production by GlcNAc requires NagS. Streptomycetes were grown as spots on R5 agar plates by plating 10 μl of 10^8^ spores ml^−1^ and after incubation at 30 °C overnight, plates were overlaid with Chrome azurol S (CAS) staining solution and examined visually. Larger orange halos show siderophore production, dark central circles are caused by the pigmented antibiotic actinorhodin. Note that the biosynthesis of siderophores and antibiotic is not repressed by GlcNAc in *nagS* mutants. CAS assays were carried out as described previously.
(TIF)

S3 Fig Roles of NagS and NagA in GlcNAc toxicity. (**a**) Suppressor mutants check for *nagB* with overexpressed *nagS* and *nagA*. Spores with different CFU of M145 complemented with empty pSET152 (M145^E^), ∆*nagB* complemented with empty pSET152 (∆*nagB*^E^), ∆*nagB* complemented with *nagS* expressed by *ermE* (∆*nagB*-*nagS*^OE^), and ∆*nagB* complemented with *nagA* expressed by *ermE* (∆*nagB*-*nagA*^OE^) were streaked on the MM supplemented with 1% mannitol and 10 mM GlcNAc. After 72 h-culturing, the numbers of suppressor mutants were compared. (**b**) Effect of over-expression of *nagA* and *nagS* on the sensitivity of *S. coelicolor nagB* mutants to GlcNAc. Spores (5 × 10^5^ CFU) of ∆*nagB* with overexpressed *nagA* (∆*nagB*-*nagA*^OE^), ∆*nagB* with overexpressed *nagS* (∆*nagB*-*nagS*^OE^), ∆*nagB*∆*nagS* with overexpressed *nagA* (∆*nagB*∆*nagS*-*nagA*^OE^), and ∆*nagAB* with overexpressed *nagS* (∆*nagAB*-*nagS*^OE^) were streaked onto MM with 1% mannitol (Mann), or with 1% mannitol and 10 mM GlcNAc (GlcNAc). The strains were cultured for 72 h at 30 °C.
(TIF)

S4 Fig NagS is a conserved protein amongst Streptomycete organisms. (**a**) Alignment of NagS protein sequence with its homologs from other *Streptomyces* species up to residue 240. Identical amino acids are shown in dark blue, and amino acids with similar properties in light blue. The D179N mutation identified in SMA11 is indicated with the red arrow below. Residues are determined to be important for catalysis are indicated with red stars above. Alignments were analyzed by Clustal Omega and the image was generated using Jalview (Version 2.11.2.7). (**b**) Gene synteny of *nagS* (SCO4393) and its homologs in other Streptomycetaceae. Note that *nagS*-*dmdR1* is conserved in all Streptomycetaceae family except *Yinghuangia* genus. Analysis was done by SyntTax inputting NagS aa sequence and the scores are given. Homologous genes are presented in the same colors with *nagS* homologous genes indicated by the red arrow.
(TIF)

S5 Fig Size-exclusion chromatography analysis of purified NagS. Analytical gel-filtration chromatography was performed on a Superdex 200 10/300 GL column (Cytiva) equilibrated with buffer containing 20 mM HEPES pH 7.5, 300 mM NaCl, 5% glycerol, and 1 mM DTT. NagS was analyzed at two protein concentrations (35 μM and 70 μM). Elution volumes (V_e_) (S7 Table) were compared to a molecular weight (Mw) calibration curve above generated using a Protein Standard Mix under identical buffer conditions. The calibration curve followed the equation V_e_ = –1.525ln(Mw) + 20.659 (R² = 0.964). Note: NagS eluted at 14.45 ml, which corresponds to an estimated molecular weight of ~60.04 kDa according to the calibration curve equation. Given that the calculated molecular weight of a NagS-His₆ monomer is 28.29 kDa, these results indicate that NagS exists as a dimer in the tested buffer. The data underlying this Figure can be found in S1 Data.
(TIF)

S6 Fig Catalytic substrates of NagS. (**a**) Initial ITC study of NagS. For ITC binding studies, 1 mM ligand was titrated with 6 or 8 µL injections into 50 μM purified NagS. Fru-6P, Glc-6P, GlcN-6P, GlcNAc, GlcNAc-1P, and GlcNAc-6P were tested. Notably, NagS bound specifically to GlcNAc-6P. Absorbance changes at 230 nm were observed when 1 mM GlcNAc-6P (**b**) or ManNAc-6P (**c**) was incubated with NagS at 30 °C, whereas incubation of GlcNAc-6P with heat-inactivated NagS (**d**) served as the negative control. The data underlying this Figure can be found in S1 Data.
(TIF)

S7 Fig Function determination and kinetics of NagS. (**a**) ^1^H NMR spectrum of the enzymatic reaction mixture of GlcNAc-6P with either the active NagS (top) or the heated-inactivated one (bottom). The associated NMR peaks of the reaction product are highlighted in red circles and arrows. (**b**) HRESIMS spectrum of the NagS product, compound **1**: *m*/*z* 282.0387 [M-H]^−^ (calculated for C_8_H_13_NO_8_P, 282.0384) **(c)** ^1^H NMR spectrum of the enzymatic reaction mixture of ManNAc-6P with either the active NagS (top) or the heat-inactivated one (bottom). The associated NMR peaks of the reaction product are highlighted in red circles. (**d**) Reactions catalyzed by NagS. NagS dehydrates both GlcNAc-6P and ManNAc-6P to produce compound **1**. Michaelis-Menten curves were fitted, and selected curves are shown for NagS with the substrates GlcNAc-6P (**e**) with *K*_m_ value of 0.45 ± 0.03 mM and *k*_cat_/*K*_m_ value of 5.48 × 10^4^ M^−1^·s^−1^, and ManNAc-6P (**f**) with *K*_m_ value of 0.68 ± 0.05 mM and *k*_cat_/*K*_m_ value of 1.32 × 10^3^ M^−1^·s^−1^. In **e** and **f**, the *V*_0_ data were plotted against the substrate concentration, and each assay was performed in triplicate and expressed as a mean ± standard error. The data underlying this Figure can be found in S1 Data.
(TIF)

S8 Fig Spectroscopic data of compound 1. (**a**) ^1^H NMR spectrum of **1** in the reaction mixture (600 MHz, in D_2_O). Nonoverlapping peaks are integrated. (**b**) ^13^C NMR spectrum of **1** in the reaction mixture (213 MHz, in D_2_O). (**c**) Multiplicity-edited HSQC spectrum of **1** in the reaction mixture (600 MHz, in D_2_O). (**d**) HMBC spectrum of **1** in the reaction mixture (600 MHz, in D_2_O). (**e**) COSY spectrum of **1** in the reaction mixture (600 MHz, in D_2_O). (**f**) TOCSY spectrum of **1** in the reaction mixture (600 MHz, in D_2_O). (**g**) NOESY spectrum of **1** in the reaction mixture (600 MHz, in D_2_O).
(TIF)

S9 Fig Activity test of NagS homologs in vitro and in vivo. **(a)** Comparison of amino acid sequences of *S. coelicolor* NagS and its homologs. These are homologs from *Streptacidiphilus jiangxiensis* (TrEMBL A0A1H7F721), *Clostridium amylolyticum* (TrEMBL A0A1M6IM34), *Paenibacillus selenitireducens* (TrEMBL A0A1T2XKX6), and *Acidothermus cellulolyticus* (TrEMBL A0LSD9). The amino acid identities with *S. coelicolor* NagS are 65.5%, 33.2%, 25.7%, and 35.7%, respectively. Absorbance changes detected at 230 nm when incubating 2 mM GlcNAc-6P with proteins from *S. jiangxiensis* **(b)**, *C. amylolyticum* **(c)**, *P. selenitireducens* **(d),** and *A. cellulolyticus* **(e)** at 30 °C. Increased absorbance means that GlcNAc-6P was dehydrated by the incubated NagS homologs. This shows that only the protein from *S. jiangxiensis* is a true NagS homolog. **(f)** In vivo activity test of NagS homologs. GlcNAc sensitivity of ∆*nagB*∆*nagS* harboring clones expressing the homologs from *S. jiangxiensis* (A0A1H7F721), *C. amylolyticum* (A0A1M6IM34), *P. selenitireducens* (A0A1T2XKX6), and *A. cellulolyticus* (A0LSD9) were grown on MM agar supplemented with 1% mannitol (Mann) and 1% mannitol plus 10 mM GlcNAc (GlcNAc). As expected based on the enzymatic activities, only the complementation of A0A1H7F721 restored GlcNAc sensitivity. This supports the phylogenetic analysis that true NagS orthologs are only found in *Streptomycetaceae*. The data underlying this Figure can be found in S1 Data.
(TIF)

S10 Fig Inhibitory effect of 6-phosphogluconate (6-PG) on NagS. (**a**) Chemical structures of 6-PG and linear GlcNAc-6P. (**b**) Average *T*_m_ curve of NagS in presence of 6-PG. Melting curve of 4 µM NagS (black), 4 µM NagS with 2 mM 6-PG (large dash), 5 mM 6-PG (dot), and 10mM 6-PG (small dash). It was shown that when 6-PG concentration increases, a shift in *T*_m_ was shown. The two Tm peaks at 46.45 °C ± 0.4 and 58.0 °C ± 0.5 °C merged into a single peak at *T*_m_ 53.08 °C ± 0.03 °C in response to the addition of 10 mM 6-PG. (**c**) 6-PG detection by LC–MS. Reactions of 10 mM 6-PG with NagS (black) or deactivated NagS (red) were detected by LC-MS. Note that no 6-PG (*m*/*z* = 275.0176) was consumed in both conditions. (**d**) Evaluation of the inhibition of 6-PG on NagS activity. The activity of NagS (*V*_0_) was measured using 1 mM GlcNAc-6P as the substrate, with the addition of 0–4 mM 6-PG. (**e**) Competitive inhibition of NagS by 6-PG. The inhibition by 6-PG is presented as Lineweaver–Burk plot (*K*_i_ = 0.28 mM). (**f**) Effect of the addition of D-gluconate on GlcNAc sensitivity. Spores (5 × 10^5^ CFU) suspension of *S. coelicolor* M145 *nagB* mutant was spotted on MM supplemented with 1% mannitol, 10 mM GlcNAc and a range concentration of D-gluconate, followed by incubation for 72 h at 30 °C. The data underlying this Figure can be found in S1 Data.
(TIF)

S11 Fig Substrate binding pocket and the movable loop of NagS. **(a)** Substrate binding pocket of NagS located at the dimeric interface. (**b**) Secondary structure alignment of ligand-free (white) and ligand-bound (cyan) NagS. The substrate GlcNAc-6P is shown in the form of sphere. The direction and angle of movement of this loop after binding the substrate is indicated by the red arrow. Note that this loop moves towards the bound substrate after binding to it. **(c)** Cross-eyed stereo view of the ordered water molecules located at the GlcNAc-6P binding site.
(TIF)

S12 Fig Kinetics of NagS variants. Michaelis-Menten curves were fitted, and selected curves are shown for NagS variants with the substrates GlcNAc-6P. The *V*_0_ data were plotted against the substrate concentration, and each assay was performed in triplicate and expressed as a mean ± standard error. The data underlying this Figure can be found in S1 Data.
(TIF)

S13 Fig Kinetics of *S. coelicolor* NagA and the product identification of NagS-NagA catalysis. Michaelis-Menten curves were fitted, and selected curves are shown for *S. coelicolor* NagA with the substrates: GlcNAc-6P (**a**) with *K*_m_ value of 1.59 ± 0.21 mM and *k*_cat_/*K*_m_ value of 1.01 × 10^5^ M^−1^·s^−1^, ManNAc-6P (**b**) with *K*_m_ value of 2.40 ± 0.44 mM and *k*_cat_/*K*_m_ value of 6.06 × 10^3^ M^−1^·s^−1^, and GalNAc-6P (**c**) with *K*_m_ value of 2.84 ± 0.41 mM and *k*_cat_/*K*_m_ value of 1.03 × 10^4^ M^−1^·s^−1^. (**d**) HRESIMS spectrum of the compound **2**/**3**: *m*/*z* 240.0280 [M-H] ^−^ (calculated for C_6_H_12_NO_7_P, 240.0279). In a–c, the *V*_0_ data were plotted against the substrate concentration, and each assay was performed in triplicate and expressed as a mean ± standard error. The data underlying this Figure can be found in S1 Data.
(TIF)

S14 Fig Extinction coefficient measurement of NagS. (**a**) This standard curve of GlcNAc-6P was obtained by plotting the concentration of GlcNAc-6P (1, 1.67, 2.67, 3.33, 4, and 5 mM) with the corresponding peak area detected in the LC–MS spectrum. (**b**) Area of the GlcNAc-6P peak after the reaction catalyzed by NagS. The concentration of remaining GlcNAc-6P is calculated to be 2.15 mM. The data underlying this Figure can be found in S1 Data.
(TIF)

S1 Table Bacterial strains and plasmids used in this study. (PDF)

S2 Table Primers used in this study. (PDF)

S3 Table NagS data collection and model refinement statistics. (PDF)

S4 Table NagS BLASTP hits in StringDB v 12. (PDF)

S5 Table NMR data of 1 as compared to Chromogen I. (PDF)

S6 Table HRMS data of the compounds identified in this study. (PDF)

S7 Table Molecular weight standards used for size-exclusion chromatography calibration. (PDF)

S1 Data Data underlying figures. (XLSX)

## Abbreviations

6-PG: 6-phosphogluconate
BGCs: biosynthetic gene clusters
CFU: colony forming units
Fru-6P: fructose-6P
GlcN-6P: glucosamine-6-phosphate
GlcNAc: N-acetylglucosamine
GlmS: glutamine-fructose-6-phosphate aminotransferase
ITC: isothermal titration calorimetry
LC–MS: Liquid Chromatography–Mass Spectrometry
MM: minimal media
MurQ: N-acetylmuramic acid-6-phosphate etherase
NagS: GlcNAc-6P dehydratase
NMR: nuclear magnetic resonance
PCD: programmed cell death
Pgi: phosphoglucose isomerase
PPP: pentose phosphate pathway
PTS: phosphotransferase system
SFM: soy flour mannitol
SIS: sugar isomerase
SNP: single nucleotide polymorphism.

## References

[1] Claessen D, Rozen DE, Kuipers OP, Søgaard-Andersen L, van Wezel GP. Bacterial solutions to multicellularity: a tale of biofilms, filaments and fruiting bodies. Nat Rev Microbiol. 2014;12(2):115–24. doi: 10.1038/nrmicro3178 24384602

[2] Hopwood DA. *Streptomyces* in nature and medicine: the antibiotic makers. Oxford University Press; 2007.

[3] Bérdy J. Thoughts and facts about antibiotics: where we are now and where we are heading. J Antibiot (Tokyo). 2012;65(8):385–95. doi: 10.1038/ja.2012.27 22511224

[4] Barka EA, Vatsa P, Sanchez L, Gaveau-Vaillant N, Jacquard C, Meier-Kolthoff JP, et al. Taxonomy, physiology, and natural products of actinobacteria. Microbiol Mol Biol Rev. 2015;80(1):1–43. doi: 10.1128/MMBR.00019-15 26609051 PMC4711186

[5] Merrick MJ. A morphological and genetic mapping study of bald colony mutants of *Streptomyces coelicolor*. J Gen Microbiol. 1976;96(2):299–315. doi: 10.1099/00221287-96-2-299 186556

[6] Nodwell JR, Yang M, Kuo D, Losick R. Extracellular complementation and the identification of additional genes involved in aerial mycelium formation in *Streptomyces coelicolor*. Genetics. 1999;151(2):569–84. doi: 10.1093/genetics/151.2.569 9927452 PMC1460480

[7] Willey J, Santamaria R, Guijarro J, Geistlich M, Losick R. Extracellular complementation of a developmental mutation implicates a small sporulation protein in aerial mycelium formation by *S. coelicolor*. Cell. 1991;65(4):641–50. doi: 10.1016/0092-8674(91)90096-h 2032288

[8] Kodani S, Hudson ME, Durrant MC, Buttner MJ, Nodwell JR, Willey JM. The SapB morphogen is a lantibiotic-like peptide derived from the product of the developmental gene *ramS* in *Streptomyces coelicolor*. Proc Natl Acad Sci U S A. 2004;101(31):11448–53. doi: 10.1073/pnas.0404220101 15277670 PMC509221

[9] Hopwood DA, Wildermuth H, Palmer HM. Mutants of *Streptomyces coelicolor* defective in sporulation. J Gen Microbiol. 1970;61(3):397–408. doi: 10.1099/00221287-61-3-397 4922764

[10] Ryding NJ, Bibb MJ, Molle V, Findlay KC, Chater KF, Buttner MJ. New sporulation loci in *Streptomyces coelicolor* A3(2). J Bacteriol. 1999;181(17):5419–25. doi: 10.1128/JB.181.17.5419-5425.1999 10464216 PMC94051

[11] Bibb M. 1995 Colworth Prize Lecture. The regulation of antibiotic production in *Streptomyces coelicolor* A3(2). Microbiology (Reading). 1996;142(Pt 6):1335–44. doi: 10.1099/13500872-142-6-1335 8704973

[12] van der Heul HU, Bilyk BL, McDowall KJ, Seipke RF, van Wezel GP. Regulation of antibiotic production in Actinobacteria: new perspectives from the post-genomic era. Nat Prod Rep. 2018;35(6):575–604. doi: 10.1039/c8np00012c 29721572

[13] Willemse J, Borst JW, de Waal E, Bisseling T, van Wezel GP. Positive control of cell division: FtsZ is recruited by SsgB during sporulation of *Streptomyces*. Genes Dev. 2011;25(1):89–99. doi: 10.1101/gad.600211 21205868 PMC3012939

[14] Willemse J, Mommaas AM, van Wezel GP. Constitutive expression of *ftsZ* overrides the *whi* developmental genes to initiate sporulation of *Streptomyces coelicolor*. Antonie Van Leeuwenhoek. 2012;101(3):619–32. doi: 10.1007/s10482-011-9678-7 22113698 PMC3278627

[15] Falguera JVT, Stratton KJ, Bush MJ, Jani C, Findlay KC, Nodwell JR, et al. DNA damage-induced block of sporulation in *Streptomyces venezuelae* involves downregulation of *ssgB*. Microbiology (Reading). 2022;168(6). 10.1099/mic.0.001198 35704023

[16] Manteca Á, Fernández M, Sánchez J. A death round affecting a young compartmentalized mycelium precedes aerial mycelium dismantling in confluent surface cultures of *Streptomyces antibioticus*. Microbiology (Reading). 2005;151(Pt 11):3689–97. doi: 10.1099/mic.0.28045-0 16272390

[17] Tenconi E, Traxler MF, Hoebreck C, van Wezel GP, Rigali S. Production of prodiginines is part of a programmed cell death process in *Streptomyces coelicolor*. Front Microbiol. 2018;9:1742. doi: 10.3389/fmicb.2018.01742 30127771 PMC6087738

[18] Rigali S, Titgemeyer F, Barends S, Mulder S, Thomae AW, Hopwood DA, et al. Feast or famine: the global regulator DasR links nutrient stress to antibiotic production by *Streptomyces*. EMBO Rep. 2008;9(7):670–5. doi: 10.1038/embor.2008.83 18511939 PMC2475330

[19] Rigali S, Nothaft H, Noens EEE, Schlicht M, Colson S, Müller M, et al. The sugar phosphotransferase system of *Streptomyces coelicolor* is regulated by the GntR-family regulator DasR and links *N*-acetylglucosamine metabolism to the control of development. Mol Microbiol. 2006;61(5):1237–51. doi: 10.1111/j.1365-2958.2006.05319.x 16925557

[20] Craig M, Lambert S, Jourdan S, Tenconi E, Colson S, Maciejewska M, et al. Unsuspected control of siderophore production by *N*-acetylglucosamine in streptomycetes. Environ Microbiol Rep. 2012;4(5):512–21. doi: 10.1111/j.1758-2229.2012.00354.x 23760896

[21] Lambert S, Traxler MF, Craig M, Maciejewska M, Ongena M, van Wezel GP, et al. Altered desferrioxamine-mediated iron utilization is a common trait of bald mutants of *Streptomyces coelicolor*. Metallomics. 2014;6(8):1390–9. doi: 10.1039/c4mt00068d 24788337

[22] Świątek-Połatyńska MA, Bucca G, Laing E, Gubbens J, Titgemeyer F, Smith CP, et al. Genome-wide analysis of in vivo binding of the master regulator DasR in *Streptomyces coelicolor* identifies novel non-canonical targets. PLoS One. 2015;10(4):e0122479. doi: 10.1371/journal.pone.0122479 25875084 PMC4398421

[23] van Bergeijk DA, Terlouw BR, Medema MH, van Wezel GP. Ecology and genomics of Actinobacteria: new concepts for natural product discovery. Nat Rev Microbiol. 2020;18(10):546–58. doi: 10.1038/s41579-020-0379-y 32483324

[24] Gavriilidou A, Kautsar SA, Zaburannyi N, Krug D, Müller R, Medema MH, et al. Compendium of specialized metabolite biosynthetic diversity encoded in bacterial genomes. Nat Microbiol. 2022;7(5):726–35. doi: 10.1038/s41564-022-01110-2 35505244

[25] Urem M, Świątek-Połatyńska MA, Rigali S, van Wezel GP. Intertwining nutrient-sensory networks and the control of antibiotic production in *Streptomyces*. Mol Microbiol. 2016;102(2):183–95. doi: 10.1111/mmi.13464 27425419

[26] Rigali S, Anderssen S, Naome A, van Wezel GP. Cracking the regulatory code of biosynthetic gene clusters as a strategy for natural product discovery. Biochem Pharmacol. 2018. 10.1016/j.bcp.2018.01.007 29309762

[27] Nothaft H, Dresel D, Willimek A, Mahr K, Niederweis M, Titgemeyer F. The phosphotransferase system of *Streptomyces coelicolor* is biased for *N*-acetylglucosamine metabolism. J Bacteriol. 2003;185(23):7019–23. doi: 10.1128/JB.185.23.7019-7023.2003 14617669 PMC262694

[28] den Hengst CD, Tran NT, Bibb MJ, Chandra G, Leskiw BK, Buttner MJ. Genes essential for morphological development and antibiotic production in *Streptomyces coelicolor* are targets of BldD during vegetative growth. Mol Microbiol. 2010;78(2):361–79. doi: 10.1111/j.1365-2958.2010.07338.x 20979333

[29] Tschowri N, Schumacher MA, Schlimpert S, Chinnam NB, Findlay KC, Brennan RG, et al. Tetrameric c-di-GMP mediates effective transcription factor dimerization to control *Streptomyces* development. Cell. 2014;158(5):1136–47. doi: 10.1016/j.cell.2014.07.022 25171413 PMC4151990

[30] Świątek MA, Urem M, Tenconi E, Rigali S, van Wezel GP. Engineering of *N*-acetylglucosamine metabolism for improved antibiotic production in *Streptomyces coelicolor* A3(2) and an unsuspected role of NagA in glucosamine metabolism. Bioengineered. 2012;3(5):280–5. doi: 10.4161/bioe.21371 22892576 PMC3477696

[31] Li C, Urem M, Du C, Zhang L, van Wezel GP. Systems-wide analysis of the ROK-family regulatory gene rokL6 and its role in the control of glucosamine toxicity in *Streptomyces coelicolor*. Appl Environ Microbiol. 2023;89(12):e0167423. doi: 10.1128/aem.01674-23 37982622 PMC10734537

[32] Bateman A. The SIS domain: a phosphosugar-binding domain. Trends Biochem Sci. 1999;24(3):94–5. doi: 10.1016/s0968-0004(99)01357-2 10203754

[33] Jaeger T, Mayer C. *N*-acetylmuramic acid 6-phosphate lyases (MurNAc etherases): role in cell wall metabolism, distribution, structure, and mechanism. Cell Mol Life Sci. 2008;65(6):928–39. doi: 10.1007/s00018-007-7399-x 18049859 PMC11131651

[34] Kim Y, Quartey P, Ng R, Zarembinski TI, Joachimiak A. Crystal structure of YfeU protein from *Haemophilus influenzae*: a predicted etherase involved in peptidoglycan recycling. J Struct Funct Genomics. 2009;10(2):151–6. doi: 10.1007/s10969-009-9063-1 19234762 PMC2771635

[35] Reith J, Mayer C. Characterization of a glucosamine/glucosaminide *N*-acetyltransferase of *Clostridium acetobutylicum*. J Bacteriol. 2011;193(19):5393–9. doi: 10.1128/JB.05519-11 21784938 PMC3187384

[36] Song Y, Huang H, Chen Y, Ding J, Zhang Y, Sun A, et al. Cytotoxic and antibacterial marfuraquinocins from the deep South China Sea-derived *Streptomyces niveus* SCSIO 3406. J Nat Prod. 2013;76(12):2263–8. doi: 10.1021/np4006025 24251399

[37] Zeyhle P, Bauer JS, Steimle M, Leipoldt F, Rösch M, Kalinowski J, et al. A membrane-bound prenyltransferase catalyzes the O-prenylation of 1,6-dihydroxyphenazine in the marine bacterium *Streptomyces* sp. CNQ-509. Chembiochem. 2014;15(16):2385–92. doi: 10.1002/cbic.201402394 25224759

[38] Szklarczyk D, Kirsch R, Koutrouli M, Nastou K, Mehryary F, Hachilif R, et al. The STRING database in 2023: protein-protein association networks and functional enrichment analyses for any sequenced genome of interest. Nucleic Acids Res. 2023;51(D1):D638–46. doi: 10.1093/nar/gkac1000 36370105 PMC9825434

[39] Tunca S, Barreiro C, Sola-Landa A, Coque JJR, Martín JF. Transcriptional regulation of the desferrioxamine gene cluster of *Streptomyces coelicolor* is mediated by binding of DmdR1 to an iron box in the promoter of the *desA* gene. FEBS J. 2007;274(4):1110–22. doi: 10.1111/j.1742-4658.2007.05662.x 17257267

[40] Augustijn HE, Reitz ZL, Zhang L, Boot JA, Elsayed SS, Challis GL, et al. Genome mining based on transcriptional regulatory networks uncovers a novel locus involved in desferrioxamine biosynthesis. PLoS Biol. 2025;23(6):e3003183. doi: 10.1371/journal.pbio.3003183 40504771 PMC12161575

[41] Chiku K, Nishimoto M, Kitaoka M. Thermal decomposition of β-D-galactopyranosyl-(1→ 3)-2-acetamido-2-deoxy-D-hexopyranoses under neutral conditions. Carbohydr Res. 2010;345(13):1901–8. doi: 10.1016/j.carres.2010.06.003 20630500

[42] Taylor PL, Blakely KM, de Leon GP, Walker JR, McArthur F, Evdokimova E, et al. Structure and function of sedoheptulose-7-phosphate isomerase, a critical enzyme for lipopolysaccharide biosynthesis and a target for antibiotic adjuvants. J Biol Chem. 2008;283(5):2835–45. doi: 10.1074/jbc.M706163200 18056714

[43] Hadi T, Hazra S, Tanner ME, Blanchard JS. Structure of MurNAc 6-phosphate hydrolase (MurQ) from *Haemophilus influenzae* with a bound inhibitor. Biochemistry. 2013;52(51):9358–66. doi: 10.1021/bi4010446 24251551 PMC3898461

[44] Souza JM, Plumbridge JA, Calcagno ML. *N*-acetylglucosamine-6-phosphate deacetylase from *Escherichia coli*: purification and molecular and kinetic characterization. Arch Biochem Biophys. 1997;340(2):338–46. doi: 10.1006/abbi.1997.9780 9143339

[45] Ahangar MS, Furze CM, Guy CS, Cooper C, Maskew KS, Graham B, et al. Structural and functional determination of homologs of the *Mycobacterium tuberculosis N*-acetylglucosamine-6-phosphate deacetylase (NagA). J Biol Chem. 2018;293(25):9770–83. doi: 10.1074/jbc.RA118.002597 29728457 PMC6016474

[46] Solomons JTG, Zimmerly EM, Burns S, Krishnamurthy N, Swan MK, Krings S, et al. The crystal structure of mouse phosphoglucose isomerase at 1.6A resolution and its complex with glucose 6-phosphate reveals the catalytic mechanism of sugar ring opening. J Mol Biol. 2004;342(3):847–60. doi: 10.1016/j.jmb.2004.07.085 15342241

[47] Hulst MB, Grocholski T, Neefjes JJC, van Wezel GP, Metsä-Ketelä M. Anthracyclines: biosynthesis, engineering and clinical applications. Nat Prod Rep. 2022;39(4):814–41. doi: 10.1039/d1np00059d 34951423

[48] Sambrook J, Fritsch EF, Maniatis T. Molecular cloning: a laboratory manual. Cold Spring Harbor Laboratory Press; 1989.

[49] MacNeil DJ, Gewain KM, Ruby CL, Dezeny G, Gibbons PH, MacNeil T. Analysis of *Streptomyces avermitilis* genes required for avermectin biosynthesis utilizing a novel integration vector. Gene. 1992;111(1):61–8. doi: 10.1016/0378-1119(92)90603-m 1547955

[50] Kieser T, Bibb MJ, Buttner MJ, Chater KF, Hopwood DA. Practical *Streptomyces* genetics. Norwich: John Innes Foundation; 2000.

[51] Hoskisson PA, van Wezel GP. Streptomyces coelicolor. Trends Microbiol. 2019;27(5):468–9. doi: 10.1016/j.tim.2018.12.008 30621999

[52] Bierman M, Logan R, O’Brien K, Seno ET, Rao RN, Schoner BE. Plasmid cloning vectors for the conjugal transfer of DNA from *Escherichia coli* to *Streptomyces* spp. Gene. 1992;116(1):43–9. doi: 10.1016/0378-1119(92)90627-2 1628843

[53] Vara J, Lewandowska-Skarbek M, Wang YG, Donadio S, Hutchinson CR. Cloning of genes governing the deoxysugar portion of the erythromycin biosynthesis pathway in *Saccharopolyspora erythraea* (*Streptomyces erythreus*). J Bacteriol. 1989;171(11):5872–81. doi: 10.1128/jb.171.11.5872-5881.1989 2681144 PMC210448

[54] van Wezel GP, Mahr K, König M, Traag BA, Pimentel-Schmitt EF, Willimek A, et al. GlcP constitutes the major glucose uptake system of *Streptomyces coelicolor* A3(2). Mol Microbiol. 2005;55(2):624–36. doi: 10.1111/j.1365-2958.2004.04413.x 15659175

[55] Fedoryshyn M, Welle E, Bechthold A, Luzhetskyy A. Functional expression of the Cre recombinase in actinomycetes. Appl Microbiol Biotechnol. 2008;78(6):1065–70. doi: 10.1007/s00253-008-1382-9 18299828

[56] Świątek MA, Tenconi E, Rigali S, van Wezel GP. Functional analysis of the *N*-acetylglucosamine metabolic genes of *Streptomyces coelicolor* and role in control of development and antibiotic production. J Bacteriol. 2012;194(5):1136–44. doi: 10.1128/JB.06370-11 22194457 PMC3294797

[57] Khodakaramian G, Lissenden S, Gust B, Moir L, Hoskisson PA, Chater KF, et al. Expression of Cre recombinase during transient phage infection permits efficient marker removal in *Streptomyces*. Nucleic Acids Res. 2006;34(3):e20. doi: 10.1093/nar/gnj019 16473843 PMC1363781

[58] Gibson DG, Young L, Chuang R-Y, Venter JC, Hutchison CA 3rd, Smith HO. Enzymatic assembly of DNA molecules up to several hundred kilobases. Nat Methods. 2009;6(5):343–5. doi: 10.1038/nmeth.1318 19363495

[59] Motamedi H, Shafiee A, Cai SJ. Integrative vectors for heterologous gene expression in *Streptomyces* spp. Gene. 1995;160(1):25–31. doi: 10.1016/0378-1119(95)00191-8 7628712

[60] Ferreira FM, Mendoza-Hernandez G, Castañeda-Bueno M, Aparicio R, Fischer H, Calcagno ML, et al. Structural analysis of *N*-acetylglucosamine-6-phosphate deacetylase apoenzyme from *Escherichia coli*. J Mol Biol. 2006;359(2):308–21. doi: 10.1016/j.jmb.2006.03.024 16630633

[61] Hall RS, Xiang DF, Xu C, Raushel FM. *N*-Acetyl-D-glucosamine-6-phosphate deacetylase: substrate activation via a single divalent metal ion. Biochemistry. 2007;46(27):7942–52. doi: 10.1021/bi700543x 17567047 PMC2533526

[62] Altschul SF, Wootton JC, Gertz EM, Agarwala R, Morgulis A, Schäffer AA, et al. Protein database searches using compositionally adjusted substitution matrices. FEBS J. 2005;272(20):5101–9. doi: 10.1111/j.1742-4658.2005.04945.x 16218944 PMC1343503

[63] Tamura K, Stecher G, Kumar S. MEGA11: molecular evolutionary genetics analysis version 11. Mol Biol Evol. 2021;38(7):3022–7. doi: 10.1093/molbev/msab120 33892491 PMC8233496

[64] Oberto J. SyntTax: a web server linking synteny to prokaryotic taxonomy. BMC Bioinformatics. 2013;14:4. doi: 10.1186/1471-2105-14-4 23323735 PMC3571937

[65] Price MN, Dehal PS, Arkin AP. FastTree 2–approximately maximum-likelihood trees for large alignments. PLoS One. 2010;5(3):e9490. doi: 10.1371/journal.pone.0009490 20224823 PMC2835736

[66] Mahr K, van Wezel GP, Svensson C, Krengel U, Bibb MJ, Titgemeyer F. Glucose kinase of *Streptomyces coelicolor* A3(2): large-scale purification and biochemical analysis. Antonie Van Leeuwenhoek. 2000;78(3–4):253–61. doi: 10.1023/a:1010234916745 11386347

[67] Kabsch W. Integration, scaling, space-group assignment and post-refinement. Acta Crystallogr D Biol Crystallogr. 2010;66(Pt 2):133–44. doi: 10.1107/S0907444909047374 20124693 PMC2815666

[68] Evans PR, Murshudov GN. How good are my data and what is the resolution? Acta Crystallogr D Biol Crystallogr. 2013;69(Pt 7):1204–14. doi: 10.1107/S0907444913000061 23793146 PMC3689523

[69] Winter G, Waterman DG, Parkhurst JM, Brewster AS, Gildea RJ, Gerstel M, et al. DIALS: implementation and evaluation of a new integration package. Acta Crystallogr D Struct Biol. 2018;74(Pt 2):85–97. doi: 10.1107/S2059798317017235 29533234 PMC5947772

[70] Emsley P, Cowtan K. Coot: model-building tools for molecular graphics. Acta Crystallogr D Biol Crystallogr. 2004;60(Pt 12 Pt 1):2126–32. doi: 10.1107/S0907444904019158 15572765

[71] Emsley P, Lohkamp B, Scott WG, Cowtan K. Features and development of Coot. Acta Crystallogr D Biol Crystallogr. 2010;66(Pt 4):486–501. doi: 10.1107/S0907444910007493 20383002 PMC2852313

[72] Adams PD, Afonine PV, Bunkóczi G, Chen VB, Davis IW, Echols N, et al. PHENIX: a comprehensive Python-based system for macromolecular structure solution. Acta Crystallogr D Biol Crystallogr. 2010;66(Pt 2):213–21. doi: 10.1107/S0907444909052925 20124702 PMC2815670

[73] Lo M-C, Aulabaugh A, Jin G, Cowling R, Bard J, Malamas M, et al. Evaluation of fluorescence-based thermal shift assays for hit identification in drug discovery. Anal Biochem. 2004;332(1):153–9. doi: 10.1016/j.ab.2004.04.031 15301960

