# Supplementary figures and images for: The novel GlcNAc 6-phosphate dehydratase NagS governs a metabolic checkpoint that controls nutrient signaling in Streptomyces
Source: PLoS Biol. 2025 Nov 25;23(11):e3003514. doi: 10.1371/journal.pbio.3003514 (PMC12680351; doi:10.1371/journal.pbio.3003514)

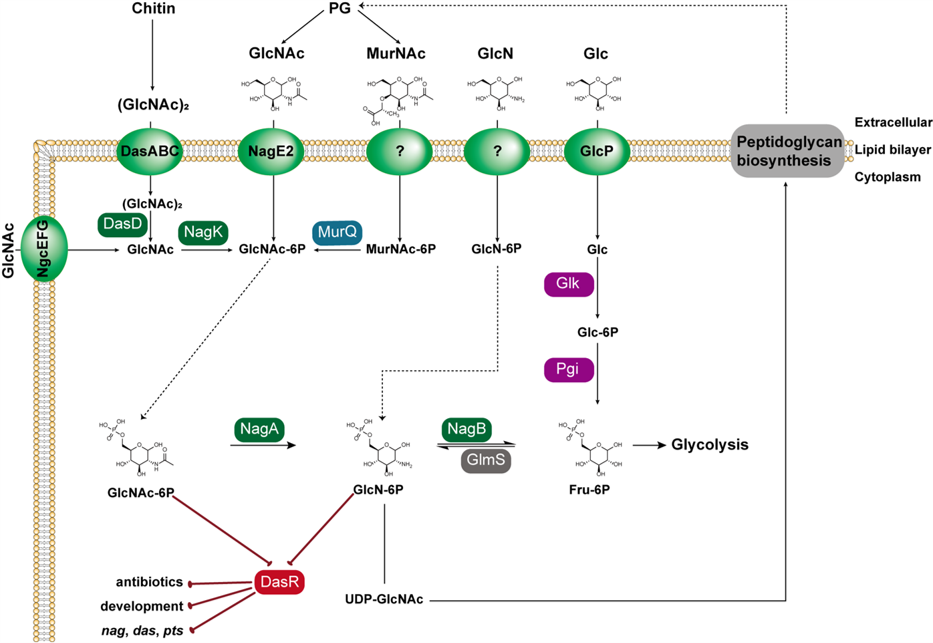

Supplement: S1 Fig — Peptidoglycan degradation releases monomers of GlcNAc and MurNAc, which are subsequently taken up by the cells for recycling. The phosphoenolpyruvate-dependent phosphotransferase system (PTS) phosphorylates monomeric GlcNAc during transport into GlcNAc-6P. Subsequently, GlcNAc-6P is metabolized by NagA and NagB to Fru-6P, which enters into glycolysis. Limited information is available on GlcN transport and metabolism in S. coelicolor. Besides its metabolism to fructose-6P, GlcN-6P is also the starting point for the biosynthesis of Lipid II, the building block for cell-wall synthesis. GlcNAc-6P and GlcN-6P are effector molecules for DasR, which is a global repressor of among others aminosugar metabolism, natural product biosynthesis, and development in Streptomyces. Metabolic routes are represented by arrows with corresponding enzymes. For clarity, the substrates and enzyme names are abbreviated. Abbreviations not mentioned in the text: Glk, glucokinase; Pgi, glucose-6-phosphate isomerase; DasD, N-acetyl-β-D-glucosaminidase. (TIF) [file pbio.3003514.s001.tif]

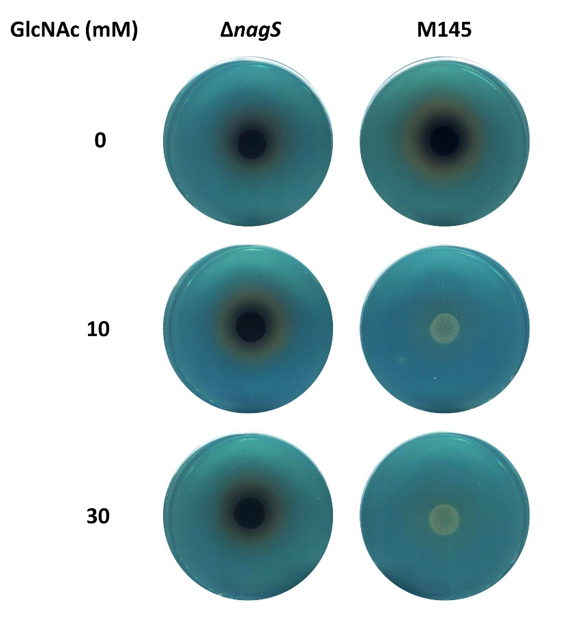

Supplement: S2 Fig — Streptomycetes were grown as spots on R5 agar plates by plating 10 μl of 108 spores ml−1 and after incubation at 30 °C overnight, plates were overlaid with Chrome azurol S (CAS) staining solution and examined visually. Larger orange halos show siderophore production, dark central circles are caused by the pigmented antibiotic actinorhodin. Note that the biosynthesis of siderophores and antibiotic is not repressed by GlcNAc in nagS mutants. CAS assays were carried out as described previously. (TIF) [file pbio.3003514.s002.tif]

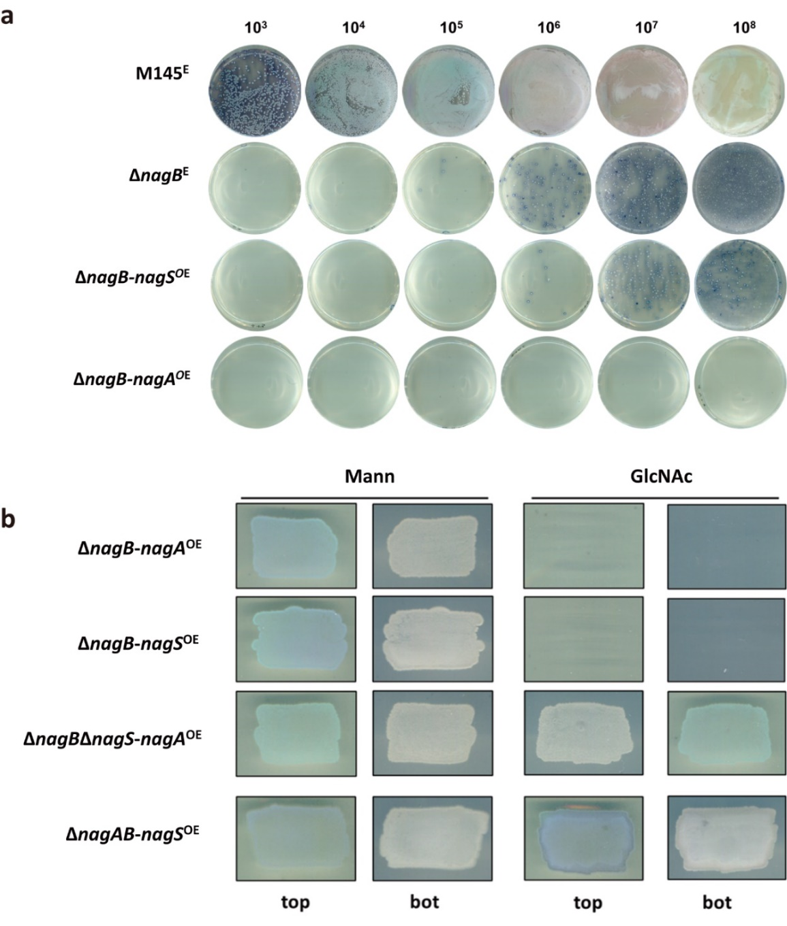

Supplement: S3 Fig — (a) Suppressor mutants check for nagB with overexpressed nagS and nagA. Spores with different CFU of M145 complemented with empty pSET152 (M145E), ∆nagB complemented with empty pSET152 (∆nagBE), ∆nagB complemented with nagS expressed by ermE (∆nagB-nagSOE), and ∆nagB complemented with nagA expressed by ermE (∆nagB-nagAOE) were streaked on the MM supplemented with 1% mannitol and 10 mM GlcNAc. After 72 h-culturing, the numbers of suppressor mutants were compared. (b) Effect of over-expression of nagA and nagS on the sensitivity of S. coelicolor nagB mutants to GlcNAc. Spores (5 × 10^5^ CFU) of ∆nagB with overexpressed nagA (∆nagB-nagAOE), ∆nagB with overexpressed nagS (∆nagB-nagSOE), ∆nagB∆nagS with overexpressed nagA (∆nagB∆nagS-nagAOE), and ∆nagAB with overexpressed nagS (∆nagAB-nagSOE) were streaked onto MM with 1% mannitol (Mann), or with 1% mannitol and 10 mM GlcNAc (GlcNAc). The strains were cultured for 72 h at 30 °C. (TIF) [file pbio.3003514.s003.tif]

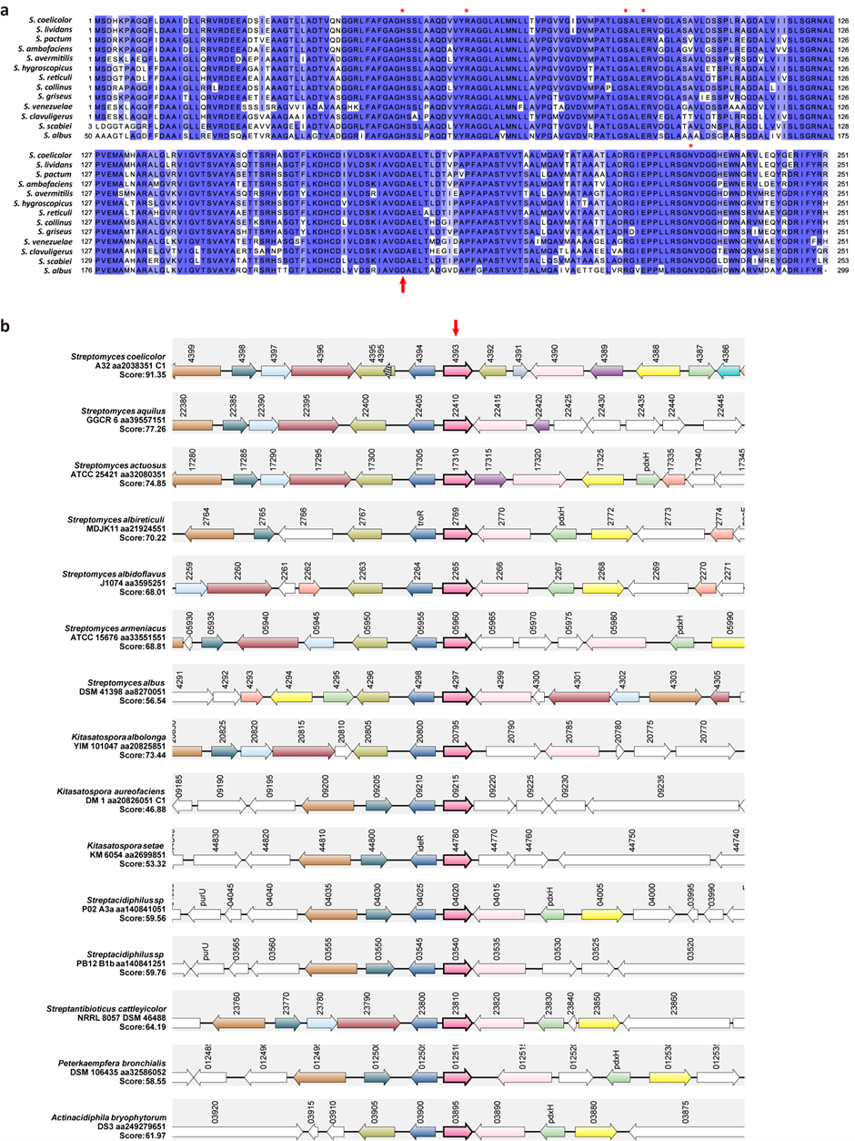

Supplement: S4 Fig — (a) Alignment of NagS protein sequence with its homologs from other Streptomyces species up to residue 240. Identical amino acids are shown in dark blue, and amino acids with similar properties in light blue. The D179N mutation identified in SMA11 is indicated with the red arrow below. Residues are determined to be important for catalysis are indicated with red stars above. Alignments were analyzed by Clustal Omega and the image was generated using Jalview (Version 2.11.2.7). (b) Gene synteny of nagS (SCO4393) and its homologs in other Streptomycetaceae. Note that nagS-dmdR1 is conserved in all Streptomycetaceae family except Yinghuangia genus. Analysis was done by SyntTax inputting NagS aa sequence and the scores are given. Homologous genes are presented in the same colors with nagS homologous genes indicated by the red arrow. (TIF) [file pbio.3003514.s004.tif]

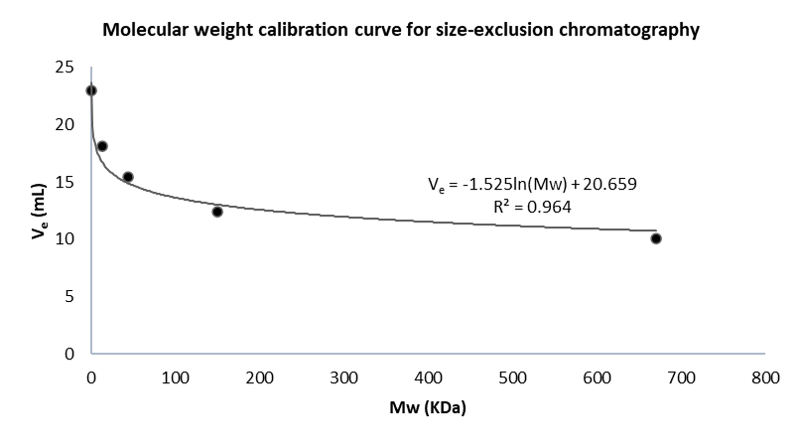

Supplement: S5 Fig — Analytical gel-filtration chromatography was performed on a Superdex 200 10/300 GL column (Cytiva) equilibrated with buffer containing 20 mM HEPES pH 7.5, 300 mM NaCl, 5% glycerol, and 1 mM DTT. NagS was analyzed at two protein concentrations (35 μM and 70 μM). Elution volumes (Ve) (S7 Table) were compared to a molecular weight (Mw) calibration curve above generated using a Protein Standard Mix under identical buffer conditions. The calibration curve followed the equation Ve = –1.525ln(Mw) + 20.659 (R² = 0.964). Note: NagS eluted at 14.45 ml, which corresponds to an estimated molecular weight of ~60.04 kDa according to the calibration curve equation. Given that the calculated molecular weight of a NagS-His₆ monomer is 28.29 kDa, these results indicate that NagS exists as a dimer in the tested buffer. The data underlying this Figure can be found in S1 Data. (TIF) [file pbio.3003514.s005.tif]

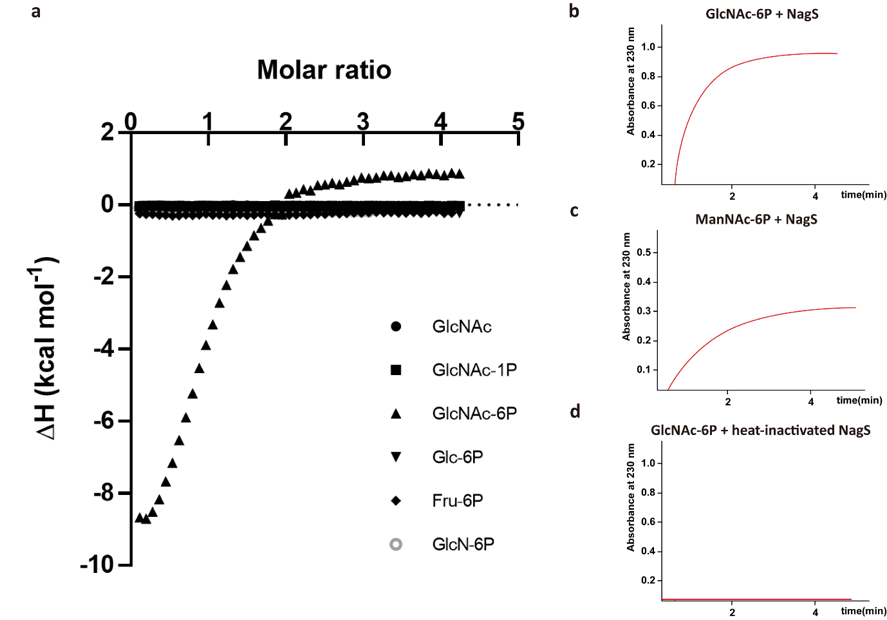

Supplement: S6 Fig — (a) Initial ITC study of NagS. For ITC binding studies, 1 mM ligand was titrated with 6 or 8 µL injections into 50 μM purified NagS. Fru-6P, Glc-6P, GlcN-6P, GlcNAc, GlcNAc-1P, and GlcNAc-6P were tested. Notably, NagS bound specifically to GlcNAc-6P. Absorbance changes at 230 nm were observed when 1 mM GlcNAc-6P (b) or ManNAc-6P (c) was incubated with NagS at 30 °C, whereas incubation of GlcNAc-6P with heat-inactivated NagS (d) served as the negative control. The data underlying this Figure can be found in S1 Data. (TIF) [file pbio.3003514.s006.tif]

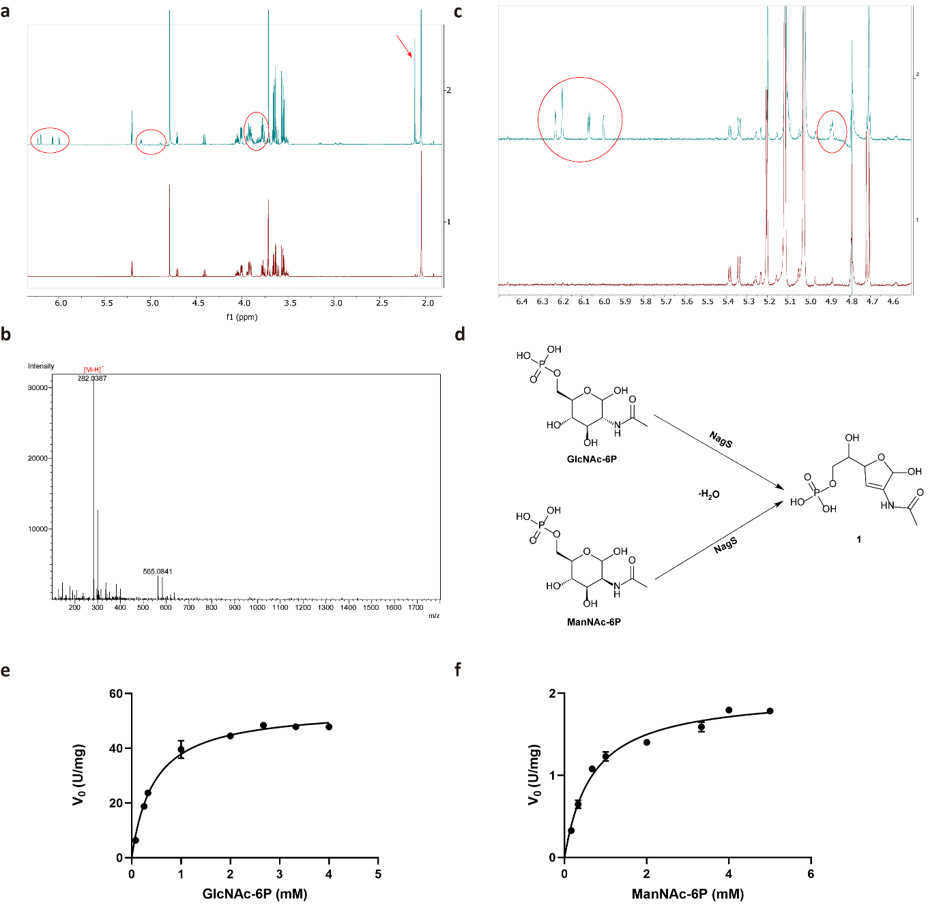

Supplement: S7 Fig — (a) 1H NMR spectrum of the enzymatic reaction mixture of GlcNAc-6P with either the active NagS (top) or the heated-inactivated one (bottom). The associated NMR peaks of the reaction product are highlighted in red circles and arrows. (b) HRESIMS spectrum of the NagS product, compound 1: m/z 282.0387 [M-H]− (calculated for C8H13NO8P, 282.0384) (c) 1H NMR spectrum of the enzymatic reaction mixture of ManNAc-6P with either the active NagS (top) or the heat-inactivated one (bottom). The associated NMR peaks of the reaction product are highlighted in red circles. (d) Reactions catalyzed by NagS. NagS dehydrates both GlcNAc-6P and ManNAc-6P to produce compound 1. Michaelis-Menten curves were fitted, and selected curves are shown for NagS with the substrates GlcNAc-6P (e) with Km value of 0.45 ± 0.03 mM and kcat/Km value of 5.48 × 10^4^ M−1·s−1, and ManNAc-6P (f) with Km value of 0.68 ± 0.05 mM and kcat/Km value of 1.32 × 10^3^ M−1·s−1. In e and f, the V0 data were plotted against the substrate concentration, and each assay was performed in triplicate and expressed as a mean ± standard error. The data underlying this Figure can be found in S1 Data. (TIF) [file pbio.3003514.s007.tif]

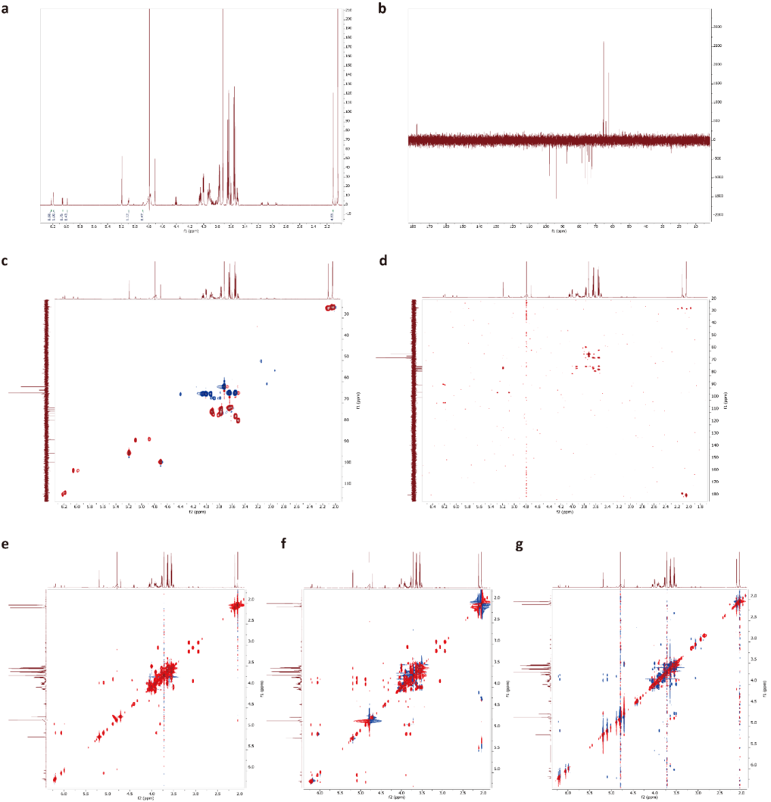

Supplement: S8 Fig — (a) 1H NMR spectrum of 1 in the reaction mixture (600 MHz, in D2O). Nonoverlapping peaks are integrated. (b) 13C NMR spectrum of 1 in the reaction mixture (213 MHz, in D2O). (c) Multiplicity-edited HSQC spectrum of 1 in the reaction mixture (600 MHz, in D2O). (d) HMBC spectrum of 1 in the reaction mixture (600 MHz, in D2O). (e) COSY spectrum of 1 in the reaction mixture (600 MHz, in D2O). (f) TOCSY spectrum of 1 in the reaction mixture (600 MHz, in D2O). (g) NOESY spectrum of 1 in the reaction mixture (600 MHz, in D2O). (TIF) [file pbio.3003514.s008.tif]

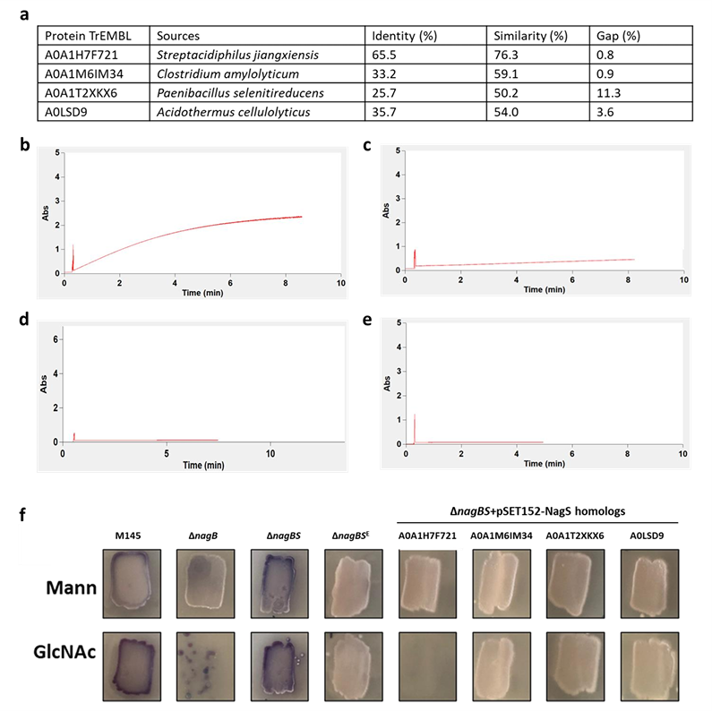

Supplement: S9 Fig — (a) Comparison of amino acid sequences of S. coelicolor NagS and its homologs. These are homologs from Streptacidiphilus jiangxiensis (TrEMBL A0A1H7F721), Clostridium amylolyticum (TrEMBL A0A1M6IM34), Paenibacillus selenitireducens (TrEMBL A0A1T2XKX6), and Acidothermus cellulolyticus (TrEMBL A0LSD9). The amino acid identities with S. coelicolor NagS are 65.5%, 33.2%, 25.7%, and 35.7%, respectively. Absorbance changes detected at 230 nm when incubating 2 mM GlcNAc-6P with proteins from S. jiangxiensis (b), C. amylolyticum (c), P. selenitireducens (d), and A. cellulolyticus (e) at 30 °C. Increased absorbance means that GlcNAc-6P was dehydrated by the incubated NagS homologs. This shows that only the protein from S. jiangxiensis is a true NagS homolog. (f) In vivo activity test of NagS homologs. GlcNAc sensitivity of ∆nagB∆nagS harboring clones expressing the homologs from S. jiangxiensis (A0A1H7F721), C. amylolyticum (A0A1M6IM34), P. selenitireducens (A0A1T2XKX6), and A. cellulolyticus (A0LSD9) were grown on MM agar supplemented with 1% mannitol (Mann) and 1% mannitol plus 10 mM GlcNAc (GlcNAc). As expected based on the enzymatic activities, only the complementation of A0A1H7F721 restored GlcNAc sensitivity. This supports the phylogenetic analysis that true NagS orthologs are only found in Streptomycetaceae. The data underlying this Figure can be found in S1 Data. (TIF) [file pbio.3003514.s009.tif]

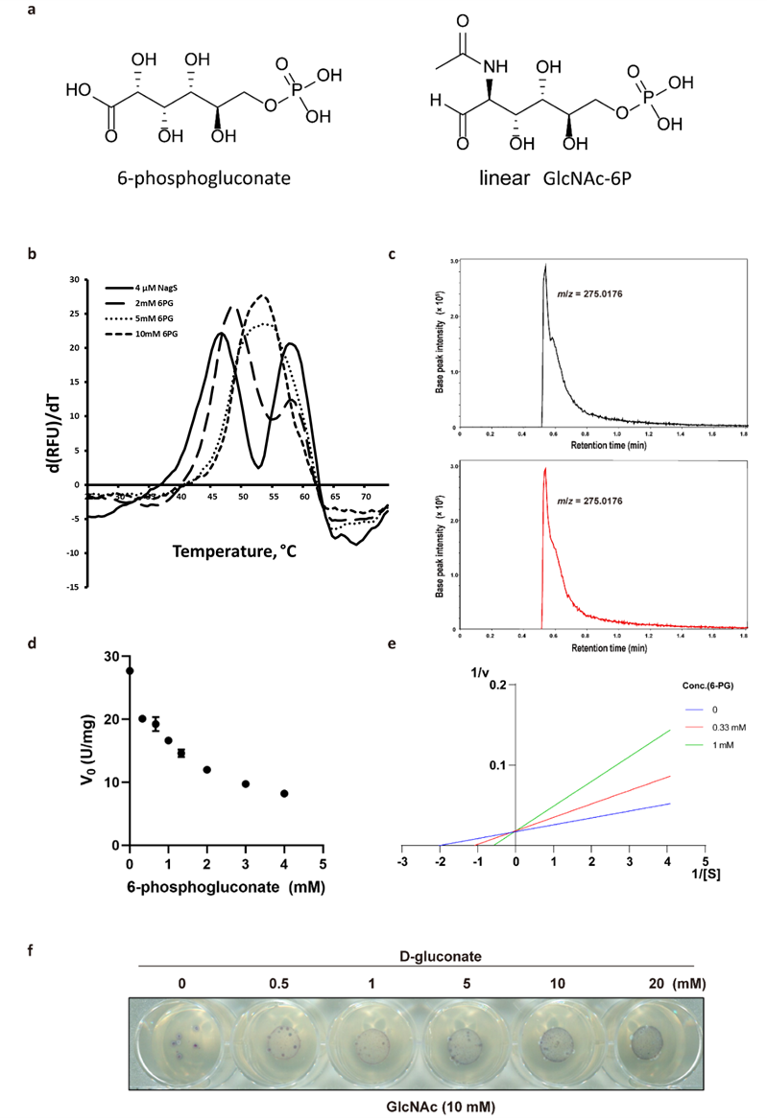

Supplement: S10 Fig — (a) Chemical structures of 6-PG and linear GlcNAc-6P. (b) Average Tm curve of NagS in presence of 6-PG. Melting curve of 4 µM NagS (black), 4 µM NagS with 2 mM 6-PG (large dash), 5 mM 6-PG (dot), and 10mM 6-PG (small dash). It was shown that when 6-PG concentration increases, a shift in Tm was shown. The two Tm peaks at 46.45 °C ± 0.4 and 58.0 °C ± 0.5 °C merged into a single peak at Tm 53.08 °C ± 0.03 °C in response to the addition of 10 mM 6-PG. (c) 6-PG detection by LC–MS. Reactions of 10 mM 6-PG with NagS (black) or deactivated NagS (red) were detected by LC-MS. Note that no 6-PG (m/z = 275.0176) was consumed in both conditions. (d) Evaluation of the inhibition of 6-PG on NagS activity. The activity of NagS (V0) was measured using 1 mM GlcNAc-6P as the substrate, with the addition of 0–4 mM 6-PG. (e) Competitive inhibition of NagS by 6-PG. The inhibition by 6-PG is presented as Lineweaver–Burk plot (Ki = 0.28 mM). (f) Effect of the addition of D-gluconate on GlcNAc sensitivity. Spores (5 × 10^5^ CFU) suspension of S. coelicolor M145 nagB mutant was spotted on MM supplemented with 1% mannitol, 10 mM GlcNAc and a range concentration of D-gluconate, followed by incubation for 72 h at 30 °C. The data underlying this Figure can be found in S1 Data. (TIF) [file pbio.3003514.s010.tif]

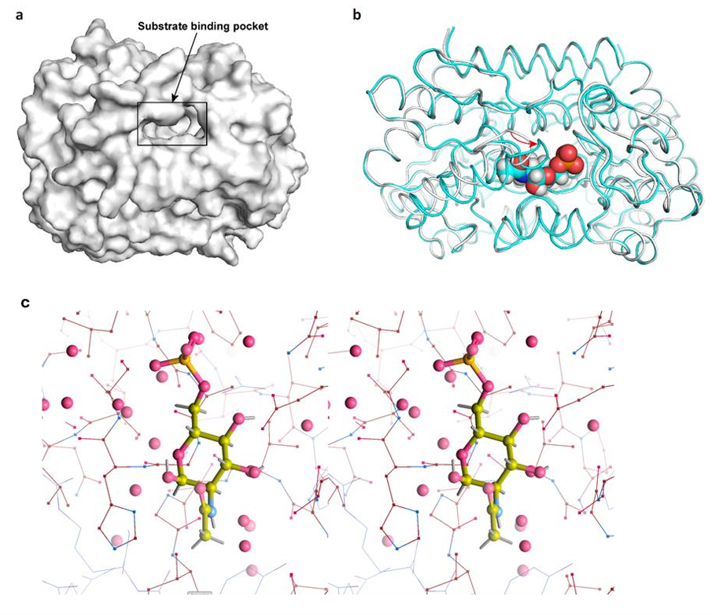

Supplement: S11 Fig — (a) Substrate binding pocket of NagS located at the dimeric interface. (b) Secondary structure alignment of ligand-free (white) and ligand-bound (cyan) NagS. The substrate GlcNAc-6P is shown in the form of sphere. The direction and angle of movement of this loop after binding the substrate is indicated by the red arrow. Note that this loop moves towards the bound substrate after binding to it. (c) Cross-eyed stereo view of the ordered water molecules located at the GlcNAc-6P binding site. (TIF) [file pbio.3003514.s011.tif]

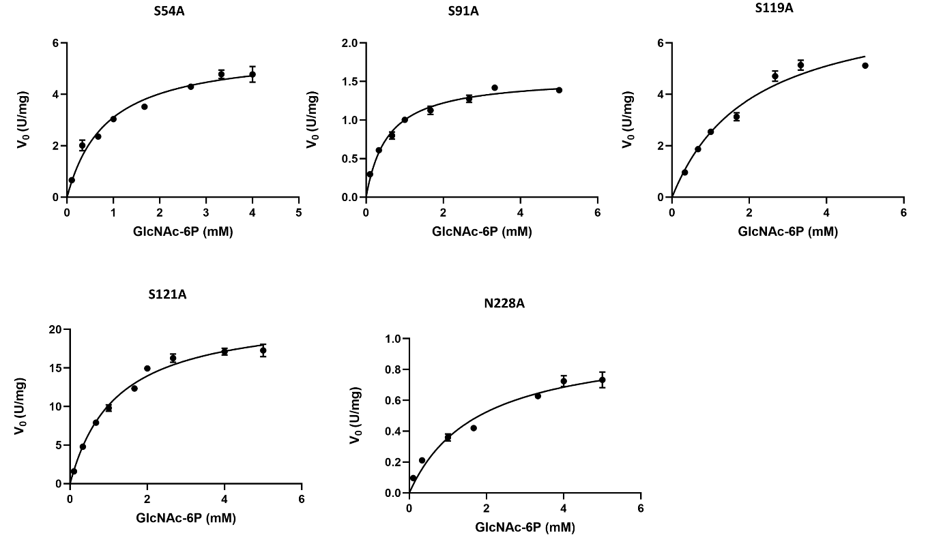

Supplement: S12 Fig — Michaelis-Menten curves were fitted, and selected curves are shown for NagS variants with the substrates GlcNAc-6P. The V0 data were plotted against the substrate concentration, and each assay was performed in triplicate and expressed as a mean ± standard error. The data underlying this Figure can be found in S1 Data. (TIF) [file pbio.3003514.s012.tif]

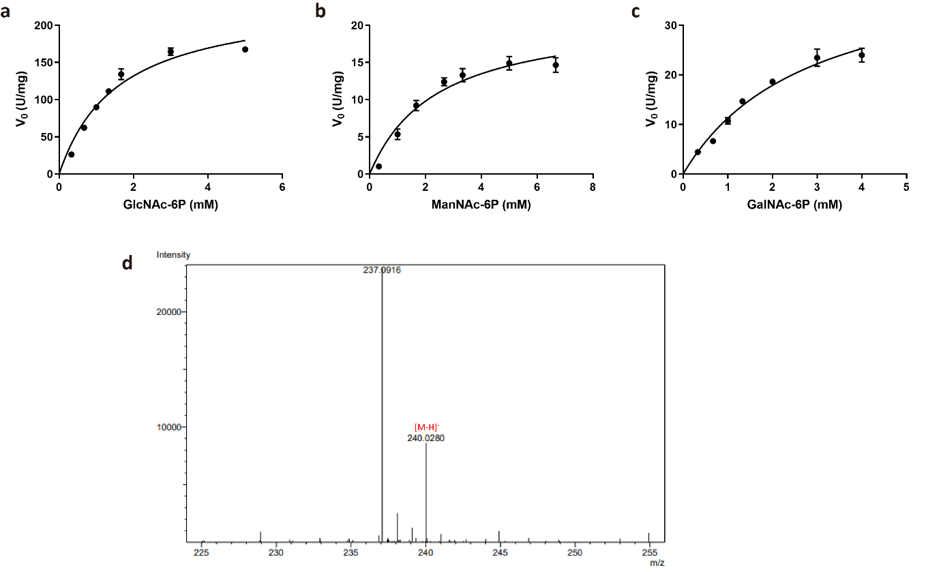

Supplement: S13 Fig — Michaelis-Menten curves were fitted, and selected curves are shown for S. coelicolor NagA with the substrates: GlcNAc-6P (a) with Km value of 1.59 ± 0.21 mM and kcat/Km value of 1.01 × 10^5^ M−1·s−1, ManNAc-6P (b) with Km value of 2.40 ± 0.44 mM and kcat/Km value of 6.06 × 10^3^ M−1·s−1, and GalNAc-6P (c) with Km value of 2.84 ± 0.41 mM and kcat/Km value of 1.03 × 10^4^ M−1·s−1. (d) HRESIMS spectrum of the compound 2/3: m/z 240.0280 [M-H] − (calculated for C6H12NO7P, 240.0279). In a–c, the V0 data were plotted against the substrate concentration, and each assay was performed in triplicate and expressed as a mean ± standard error. The data underlying this Figure can be found in S1 Data. (TIF) [file pbio.3003514.s013.tif]

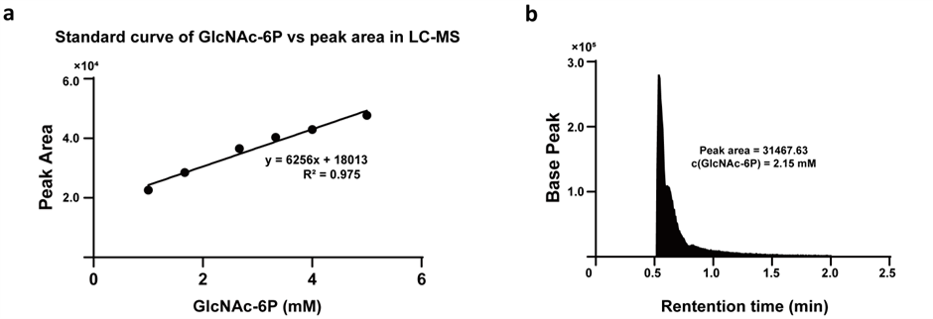

Supplement: S14 Fig — (a) This standard curve of GlcNAc-6P was obtained by plotting the concentration of GlcNAc-6P (1, 1.67, 2.67, 3.33, 4, and 5 mM) with the corresponding peak area detected in the LC–MS spectrum. (b) Area of the GlcNAc-6P peak after the reaction catalyzed by NagS. The concentration of remaining GlcNAc-6P is calculated to be 2.15 mM. The data underlying this Figure can be found in S1 Data. (TIF) [file pbio.3003514.s014.tif]
