# Supplementary material for: The novel GlcNAc 6-phosphate dehydratase NagS governs a metabolic checkpoint that controls nutrient signaling in Streptomyces
Source: PLoS Biol. 2025 Nov 25;23(11):e3003514. doi: 10.1371/journal.pbio.3003514 (PMC12680351; doi:10.1371/journal.pbio.3003514)
Supplement: S1 Table — (PDF) [file pbio.3003514.s015.pdf]

S1 Table. Bacterial strains and plasmids used in this study

Bacterial strains	Description[*#]	Reference or source
E. coli		
E. coli JM109	General cloning strain	[1]
E. coli ET12567/pUZ8002	Strain used for conjugation between *E. coli* and *Streptomyces*	[2]
E. coli Rosetta(DE3)pLysS	Strain used for protein expression	Novagen
E. coli BL21(DE3)	Strain used for protein expression	Novagen
S. coelicolor A3(2)		
M145	*S. coelicolor* A3(2) M145 SCP1- SCP2- prototroph	[3]
M145^{E}	M145 complemented with empty pSET152	This work
ΔnagB	M145 Δ*nagB*^{d}	[4]
ΔnagB^{E}	*ΔnagB* complemented with empty pSET152	This work
ΔnagAB	M145 Δ*nagB*^{d}Δ*nagA*^{d}	[4]
SMA11	M145 Δ*nagB* suppressor, mutated in *nagS*	[5]
Δ*nagS*	M145 Δ*nagS*^{d}	This work
Δ*nagB*Δ*nagS*	M145 Δ*nagB*^{d}Δ*nagS*^{d}	This work
Δ*nagB*Δ*nagS*^{C}	Δ*nagB*Δ*nagS* complemented with *nagS*	This work
Δ*nagB*Δ*nagS*$^{C-H53A}$	Δ*nagB*Δ*nagS* complemented with *nagS* with mutation H53A	This work
Δ*nagB*Δ*nagS*$^{C-S54A}$	Δ*nagB*Δ*nagS* complemented with *nagS* with mutation S54A	This work
Δ*nagB*Δ*nagS*$^{C-R64A}$	Δ*nagB*Δ*nagS* complemented with *nagS* with mutation R64A	This work
Δ*nagB*Δ*nagS*$^{C-S91A}$	Δ*nagB*Δ*nagS* complemented with *nagS* with mutation S91A	This work
Δ*nagB*Δ*nagS*$^{C-E94A}$	Δ*nagB*Δ*nagS* complemented with *nagS* with mutation E94A	This work
Δ*nagB*Δ*nagS*$^{C-S119A}$	Δ*nagB*Δ*nagS* complemented with *nagS* with mutation S119A	This work
Δ*nagB*Δ*nagS*$^{C-S121A}$	Δ*nagB*Δ*nagS* complemented with *nagS* with mutation S121A	This work
Δ*nagB*Δ*nagS*$^{C-DD179A}$	Δ*nagB*Δ*nagS* complemented with *nagS* with mutation D179A	This work
Δ*nagB*Δ*nagS*$^{C-N228A}$	Δ*nagB*Δ*nagS* complemented with *nagS* with mutation N228A	This work
Δ*nagB*Δ*nagS*^{E}	Δ*nagB*Δ*nagS* complemented with empty pSET152	This work
Δ*nagS*^{E}	Δ*nagS* complemented with empty pSET152	This work
Δ*nagB-nagS*^{OE}	Δ*nagB* with overexpressed *nagS*	This work
Δ*nagB-nagA*^{OE}	Δ*nagB* with overexpressed *nagA*	This work
Δ*nagB*Δ*nagS-nagA*^{OE}	Δ*nagB*Δ*nagS* with overexpressed *nagA*	This work
ΔnagAB-nagS^{OE}	*ΔnagAB* with overexpressed *nagS*	This work
**Plasmids**	**Description**	**Reference**
pSET152	Integrative *E. coli/Streptomyces* shuttle vector	[6]

pWHM3	*E. coli/Streptomyces* shuttle vector, high copy number and unstable in *Streptomyces*	[7]
pET15b	Vector for His_6-tagged protein overexpression in *E. coli*	Novagen
pET-28a(+)	Vector for His_6-tagged protein overexpression in *E. coli*	Novagen
pUWLcre	*E. coli/Streptomyces* shuttle vector expressing the Cre recombinase in *Streptomyces*	[8]
pCOM-4393	pSET152 harbouring *nagS* gene with its own promoter	This work
pCOM-4393-H53A	pSET152 harbouring *nagS* with the aa residue mutation H53A	This work
pCOM-4393-S54A	pSET152 harbouring *nagS* with the aa residue mutation S54A	This work
pCOM-4393-R64A	pSET152 harbouring *nagS* with the aa residue mutation R64A	This work
pCOM-4393-S91A	pSET152 harbouring *nagS* with the aa residue mutation S91A	This work
pCOM-4393-E94A	pSET152 harbouring *nagS* with the aa residue mutation E94A	This work
pCOM-4393-S119A	pSET152 harbouring *nagS* with the aa residue mutation S119A	This work
pCOM-4393-S121A	pSET152 harbouring *nagS* with the aa residue mutation S121A	This work
pCOM-4393-D179A	pSET152 harbouring *nagS* with the aa residue mutation D179A	This work
pCOM-4393-N228A	pSET152 harbouring *nagS* with the aa residue mutation N228A	This work
pOE-4393	pSET152 harbouring *nagS* gene under control of *ermE* promoter	This work
pOE-4284	pSET152 harbouring *nagA* gene under control of *ermE* promoter	This work
pKO-4393	pWHM3-oriT harbouring the *nagS* flanking regions with *loxP-aac(3)IV-loxP*	This work
pEX-4393	pET15b harbouring the *nagS* gene	This work
pEX-4393-H53A	pET15b harbouring the *nagS* with the aa residue mutation H53A	This work
pEX-4393-S54A	pET15b harbouring the *nagS* with the aa residue mutation S54A	This work
pEX-4393-R64A	pET15b harbouring the *nagS* with the aa residue mutation R64A	This work
pEX-4393-S91A	pET15b harbouring the *nagS* with the aa residue mutation S91A	This work
pEX-4393-E94A	pET15b harbouring the *nagS* with the aa residue mutation E94A	This work
pEX-4393-S119A	pET15b harbouring the *nagS* with the aa residue mutation S119A	This work
pEX-4393-S121A	pET15b harbouring the *nagS* with the aa residue mutation S121A	This work

pEX-4393-D179A	pET15b harbouring the *nagS* with the aa residue mutation D179A	This work
pEX-4393-N228A	pET15b harbouring the *nagS* with the aa residue mutation N228A	This work
pEX-4284	pET-28a(+) harbouring the gene *nagA*	This work

* "d" indicates the gene before "d" is in-frame deleted; # "::*aac(3)IV*" indicates the gene before "::"is replaced by *aac(3)IV* cassette
