# Supplementary material for: The novel GlcNAc 6-phosphate dehydratase NagS governs a metabolic checkpoint that controls nutrient signaling in Streptomyces
Source: PLoS Biol. 2025 Nov 25;23(11):e3003514. doi: 10.1371/journal.pbio.3003514 (PMC12680351; doi:10.1371/journal.pbio.3003514)
Supplement: S2 Table — (PDF) [file pbio.3003514.s016.pdf]

S2 Table. Primers used in this study

Name	5'-3' sequence*	Function or descriptions
SCO4393-LF	GTCAGAATTCACGTCGATGCGCCGCGCCATAGG	*nagS* knock-out
SCO4393-LR	GAAGTTATCCATCACCTCTAGACTTGTGGTCGCTCATGCG	*nagS* knock-out
SCO4393-RF	GAAGTTATCGCGCATCTCTAGACGCCGCTGAACGCACCCGGTG	*nagS* knock-out
SCO4393-RR	GTCAAAGCTTGCGACGCTCCATTCGAGCAGAGG	*nagS* knock-out
SCO4393-KO-CF	CGTGCCCCTCGATGAGATTG	*nagS* mutant checking
SCO4393-KO-CR	ATGTTGCGCCGCTTGTAGAAC	*nagS* mutant checking
SCO4393-compF	CTATGACATGATTACGAATTCGATTGCCCTCGTCGGTCAGCTCC	*nagS* complementation
SCO4393-compR	TGGGCTGCAGGTCGACTACTGCTTCAGGCAGGTGAAGC	*nagS* complementation
SCO4393-H53A-F	TCGCCTTCGGCGCCGGCGCCTCCTCCCTCGCCGCCCAGG	NagS-H53A PCR
SCO4393-H53A-R	GCCGGCGCCGAAGGCGAAGAG	NagS-H53A PCR
SCO4393-R64A-F	GTCGTGTACGCCGCGGGCGGGCTCGCCCTG	NagS-R64A PCR
SCO4393-R64A-R	GCCCGCGGCGTACACGACGTCCTGGGCGGCGAG	NagS-R64A PCR
SCO4393-E94A-F	CTCGGCTCCGCCCTGGCGCGGGTCGACGGCCTCGCG	NagS-E94A PCR
SCO4393-E94A-R	CCAGGGCGGAGCCGAGGGTG	NagS-E94A PCR
SCO4393-S91A-F	ATGCCGGCCACCCTCGGCGCCGCCCTGGAGCGGGTCGACG	NagS-S91A PCR
SCO4393-S91A-R	GCCGAGGGTGGCCGGCATGAC	NagS-S91A PCR
SCO4393-S119A-F	GACGCCCTGGTGATCATCGCGCTCTCCGGGCGCAACG	NagS-S119A PCR
SCO4393-S119A-R	GATGATCACCAGGGCGTCGC	NagS-S119A PCR
SCO4393-D179A-F	TCCAAGATCGCCGTCGGCGCCGCGGAACTCACCCTCGACACC	NagS-D179A PCR
SCO4393-D179A-R	GCCGACGGCGATCTTGGAGTCC	NagS-D179A PCR
SCO4393-N228A-F	CCCGCTGCTGCGCTCGGGCGCCGTGGACGGCGGCCACGAATG	NagS-N228A PCR
SCO4393-N228A-R	CGAGCGCAGCAGCGGGGGTTC	NagS-N228A PCR
SCO4393-S54A-F	CTTCGGCGCCGGCCACGCCTCCCTCGCCGCCCAGGAC	NagS-S54A PCR
SCO4393-S54A-R	GTGGCCGGCGCCGAAGGCG	NagS-S54A PCR
SCO4393-S121A-F	CCTGGTGATCATCTCGCTCGCCGGGCGCAACGCCCTGCC	NagS-S121A PCR
SCO4393-S121A-R	GAGCGAGATGATCACCAGGGCGTCG	NagS-S121A PCR
SCO4393-exp-F	GTACGAATTCATATGAGCGACCACAAGCCGGCC	NagS heterologous expression
SCO4393-exp-R	GTACGGATCCCCGGGGCACCGGGTGCGTTCA	NagS heterologous expression
NagA-exp-F	AATTTTGTTTAACTTTAAGAAGGAGATATACATGGCCCCAAGC AAGGTTCTCGC	NagA heterologous expression
NagA-exp-R	GTGGTGGTGGTGGTGCTCGCCCAGGTGGGGATCGACCAC	NagA heterologous expression
SCO4393-OE-F	ACTCCACAGGAGGACCCACAATGAGCGACCACAAGCCGGC	*nagS* overexpression
SCO4393-OE-R	GACCGAGCGTTCTGAACAAGTCAGCGGCGGTAGAAGATGCG	*nagS* overexpression
SCO4284-OE-F	CTCCACAGGAGGACCCACAATGGCCCCAAGCAAGGTTCTC	*nagA* overexpression
SCO4284-OE-R	GACCGAGCGTTCTGAACAAGTCAGCCCAGGTGGGGATCGAC	*nagA* overexpression

* Underlined characters indicating restriction sites or overhangs for Gibson assembly. GAATTC, *Eco*RI; TCTAGA, *Xba*I; AAGCTT, *Hind*III.
