# Supplementary material for: The novel GlcNAc 6-phosphate dehydratase NagS governs a metabolic checkpoint that controls nutrient signaling in Streptomyces
Source: PLoS Biol. 2025 Nov 25;23(11):e3003514. doi: 10.1371/journal.pbio.3003514 (PMC12680351; doi:10.1371/journal.pbio.3003514)
Supplement: S3 Table — (PDF) [file pbio.3003514.s017.pdf]

S3 Table. **NagS data collection and model refinement statistics**

	Apo NagS	GlcNAc-6P bound NagS	6-PG bound NagS
**Data collection**			
Space group	P 65 2 2	P 65 2 2	P 65 2 2
Cell dimensions			
a, b, c (Å)	87.69, 87.69, 273.49	88.37, 88.37, 284.46	87.67 87.67 278.81
Α, β, γ (°)	90, 90, 120	90, 90, 120	90, 90, 120
Resolution (Å)*	44.4 – 2.3 (2.38 – 2.30)	45.7 - 2.6 (2.59 – 2.68)	66.7 - 1.7 (1.72 - 1.69)
R_{meas}	0.167 (1.115)	0.256 (2.918)	0.068 (0.292)
I / σI	8.8 (1.7)	9.7 (1.2)	20.9 (1.7)
CC (1 / 2)	0.993 (0.569)	0.998 (0.527)	1.000 (0.799)
Completeness (%)	97.53 (95.97)	99.90 (99.37)	99.9 (98.6)
Multiplicity	5.3 (5.1)	18.9 (19.5)	19.7 (20.4)
**Refinement**			
Resolution (Å)	44.4 – 2.3 (2.38 – 2.3)	45.7 - 2.6 (2.71 - 2.59)	46.5 – 1.7 (1.71 – 1.69)
Number of reflections			
Used for refinement	27893 (2646)	21362 (2555)	71889 (2724)
Used for R_{free}	1380 (124)	1098 (128)	3548 (144)
R_{work}	0.181 (0.254)	0.191 (0.280)	0.180 (0.347)
R_{free}	0.214 (0.306)	0.229 (0.337)	0.205 (0.354)
non-H atoms	3887	3790	3853
NagS	3624	3626	3626
ligands	20	48	44
water	243	116	181
R.m.s deviations			
Bond lengths (Å)	0.006	0.002	0.011
Bond angles (°)	0.82	0.62	1.01
Clash score	2.2	1.9	1.4
Ramachandran			
Favored	98.0%	99.0%	99.0%
Outliers	0.3%	0.4%	0.4%
Average B-factor	35	61	45
Protein	35	61	45
Ligands	50	69	44
Water	37	54	44
PDB ID code	9F7O	9F7V	9EOL

* Values in parentheses are for highest-resolution shell.
