# Supplementary material for: The novel GlcNAc 6-phosphate dehydratase NagS governs a metabolic checkpoint that controls nutrient signaling in Streptomyces
Source: PLoS Biol. 2025 Nov 25;23(11):e3003514. doi: 10.1371/journal.pbio.3003514 (PMC12680351; doi:10.1371/journal.pbio.3003514)
Supplement: S4 Table — (PDF) [file pbio.3003514.s018.pdf]

Table S4. NagS BLASTP hits in StringDB v 12

Subject ID	aa ID (%)	length	mismatches	gaps	Expect	Bit score	Strain name
**100226.gene:17762038**	100	251	0	0	2,8E-168	472	Streptomyces coelicolor A3 2
1561022.NI25_22215	93,2	251	17	0	6,4E-158	446	Streptomyces sp CCM MD2014
68249.BC342_14275	91,2	251	22	0	5,1E-155	439	Streptomyces pactum
1915400.FM21_27680	91,2	251	22	0	5,9E-154	436	Streptomyces mutabilis
146923.Spa2297_14460	89,6	251	26	0	4E-152	431	Streptomyces parvulus
909626.AQJ91_14790	88,4	251	29	0	6,6E-151	428	Streptomyces dysideae
67356.AQJ84_19390	89,2	251	27	0	1,3E-150	427	Streptomyces resistomycificus
1917143.GCA_002005565_02350	89,2	250	27	0	1,9E-150	427	Streptomyces sp JHA26
1889.SAM40697_3035	88,4	251	29	0	1,1E-149	425	Streptomyces ambofaciens
358823.DF19_37395	89,6	250	26	0	3,1E-149	424	Streptomyces olindensis
665577.GCA_000316095_03783	88,4	251	29	0	1,3E-148	422	Streptomyces viridosporus T7A
645465.SSTG_01936	86,9	251	33	0	2,6E-148	422	Streptomyces sp e14
552354.GCA_001293595_04026	87,3	251	32	0	3,6E-148	421	Streptomyces sp TP-A0875
1079986.GCA_000226455_01721	87,6	250	31	0	5,2E-148	421	Streptomyces chartreusis NRRL 12338
1463909.GCA_000722015_04553	87,6	251	31	0	7,3E-148	421	Streptomyces sp NRRL S-474
1463861.GCA_000716605_08858	87,1	249	32	0	1E-147	420	Streptomyces sp NRRL F-525
67344.SAMN05216505_104229	87,2	250	32	0	1,5E-147	420	Streptomyces prasinopilosus
1415546.ADK57_02190	87,6	250	31	0	6E-147	418	Streptomyces sp MMG1533
**285535.GCA_000718165_00468**	86,8	250	33	0	8,5E-147	418	Streptomyces fulvoviolaceus
1352936.M878_24260	88,4	251	29	0	8,5E-147	418	Streptomyces roseochromogenus subsp oscitans DS 12 976
1855351.SAMN05216482_4296	88	250	30	0	8,5E-147	418	Streptomyces sp PAN FS17
285514.GCA_000716435_04411	85,7	251	36	0	2,4E-146	417	Streptomyces xylophagus
146922.BWZ23_17370	85,7	251	36	0	4,9E-146	416	Streptomyces griseofuscus
1570823.B7767_28970	84,9	251	38	0	9,9E-146	415	Streptomyces sp 13-12-16
1761904.SAMN04487981_102343	86,8	250	33	0	1,4E-145	415	Streptomyces sp cf386
68194.GCA_000725475_05257	87,3	251	32	0	2,9E-145	414	Streptomyces durhamensis
68214.AVL59_44545	87,1	249	32	0	2,9E-145	414	Streptomyces griseochromogenes
284040.UK15_14120	86,8	250	33	0	2,9E-145	414	Streptomyces variegatus
591159.SSQG_03468	86	250	35	0	2,9E-145	414	Streptomyces viridochromogenes DSM 40736
1214101.BN159_4958	86,1	251	35	0	2,9E-145	414	Streptomyces davaonensis JCM 4913
67386.AQI95_10970	86,5	251	34	0	4E-145	414	Streptomyces yokosukanensis
66874.GCA_000725465_02452	86,4	250	34	0	5,7E-145	413	Streptomyces bicolor
1415549.ADK65_18845	86,8	250	33	0	5,7E-145	413	Streptomyces sp NRRL B-1140
1783515.A4E84_17630	86	250	35	0	5,7E-145	413	Streptomyces qaidamensis
477245.TU94_14520	87,6	250	31	0	8,2E-145	413	Streptomyces cyaneogriseus subsp noncyanogenus
1355015.LK06_018640	86,1	251	35	0	1,2E-144	412	Streptomyces pluripotens
467200.SSRG_03584	84,4	250	39	0	1,6E-144	412	Streptomyces griseoflavus Tü4000
1840095.STXM2123_3241	85,2	250	37	0	2,3E-144	412	Streptomyces sp F-3
1849967.A8713_13525	85,3	251	37	0	2,3E-144	412	Streptomyces sp SAT1
73044.GCA_000725795_01671	85,5	249	36	0	3,3E-144	411	Streptomyces seoulensis
1577075.QR97_15245	86,8	250	33	0	3,3E-144	411	Streptomyces sp PBH53
1703942.AMK31_17850	87,2	250	32	0	3,3E-144	411	Streptomyces sp TSRI0107
55952.BU52_08195	85,9	249	35	0	4,7E-144	411	Streptomyces toyocaensis
665007.ABB07_21260	86,4	250	34	0	4,7E-144	411	Streptomyces incarnatus
1133850.SHJG_4751	86,4	250	34	0	4,7E-144	411	Streptomyces hygroscopicus subsp jinggangensis 5008
1463917.GCA_000720765_05875	85,1	249	37	0	4,7E-144	411	Streptomyces sp NRRL S-646
1463926.GCA_000718785_01568	84,4	250	39	0	4,7E-144	411	Streptomyces sp NRRL WC-3626
1855347.SAMN05216532_4754	86	250	35	0	6,7E-144	410	Streptomyces sp 2231 1
661399.AQJ67_29950	86,1	251	35	0	1,9E-143	409	Streptomyces caeruleatus
1415544.ADK64_11870	85,6	250	36	0	1,9E-143	409	Streptomyces sp MMG1121
1463901.GCA_000720185_04677	83,7	251	41	0	1,9E-143	409	Streptomyces sp NRRL S-340
1882757.SAMN05444521_5009	84,9	251	38	0	1,9E-143	409	Streptomyces sp 3214 6
1963.AQJ27_23230	85,3	251	37	0	2,7E-143	409	Streptomyces olivochromogenes
1354889.BIV25_13950	85,2	250	37	0	2,7E-143	409	Streptomyces sp MUSC 14
1463880.GCA_000720135_02411	86,5	251	34	0	3,9E-143	409	Streptomyces sp NRRL S-1022
40318.SNOD_14995	86,1	245	34	0	4,8E-143	408	Streptomyces nodosus
1968.GCA_000721105_04824	86,1	251	35	0	5,5E-143	408	Streptomyces cellulosae
164348.BFF78_23755	86,9	251	33	0	5,5E-143	408	Streptomyces puniciscabiei
710705.A6P39_31910	85,6	250	36	0	5,5E-143	408	Streptomyces sp FXJ1 172
1214242.B446_17155	85,2	250	37	0	5,5E-143	408	Streptomyces collinus Tu 365
114686.BM536_015445	84	250	40	0	7,8E-143	408	Streptomyces phaeoluteigriseus
1725411.AS200_25800	85,2	250	37	0	7,8E-143	408	Streptomyces sp CdTB01
711393.GCA_000500635_01566	85,6	250	36	0	1,1E-142	407	Streptomyces sp GXT6
1078086.HMPREF1211_06733	84,1	251	40	0	1,1E-142	407	Streptomyces sp HGB0020
1890285.GCA_001746365_01244	83,2	250	42	0	1,1E-142	407	Streptomyces sp LUP30
1955065.B0675_06990	84,5	251	39	0	1,6E-142	407	Streptomyces sp M41 2017
864058.GCA_001906585_05266	84,9	251	38	0	2,2E-142	407	Streptomyces kebangsaanensis
1577588.GCA_001417695_05088	83,2	250	42	0	2,2E-142	407	Streptomyces sp JHA19
146536.AQI70_24245	85,2	250	37	0	3,2E-142	406	Streptomyces curacoi
1855348.SAMN05216489_05822	84,7	249	38	0	3,2E-142	406	Streptomyces sp 3213 3
33903.AQJ43_05345	84,5	251	39	0	4,5E-142	406	Streptomyces avermitilis
1736452.ASD48_08675	84,8	250	38	0	4,5E-142	406	Streptomyces sp Root1310
193461.GCA_000725495_07320	83,7	251	41	0	6,4E-142	405	Streptomyces aureus
1907.SGLAU_15075	85,1	249	37	0	1,8E-141	404	Streptomyces glaucescens
68231.AQJ30_16875	83,2	250	42	0	1,8E-141	404	Streptomyces longwoodensis
1359208.GCA_001417775_01574	86,5	244	33	0	1,9E-141	405	Streptomyces sp TP-A0356
67285.AQI88_34730	84,4	250	39	0	3,7E-141	404	Streptomyces cellostaticus

67315.GCA_000718625_01992	84,5	251	39	0	3,7E-141	404	Streptomyces lavenduligriseus
67331.SAMN04490357_3668	84,5	251	39	0	5,2E-141	403	Streptomyces misionensis
285568.AQJ66_08590	84	250	40	0	5,2E-141	403	Streptomyces bungoensis
1172179.GCA_000424945_04948	84,1	251	40	0	7,4E-141	403	Streptomyces sp 142MFCol3 1
1855349.SAMN05216533_4779	85,1	249	37	0	1,1E-140	402	Streptomyces sp Ag109 O5-10
1839783.GA0115233_11138	84,4	250	39	0	2,1E-140	402	Streptomyces sp DI166
1761906.SAMN04487983_1010125	83,9	249	40	0	3E-140	401	Streptomyces sp yr375
1943.AQJ64_26960	82,8	250	43	0	9,7E-140	400	Streptomyces griseoruber
195801.GCA_000715605_01260	82,7	249	43	0	2,5E-139	399	Streptomyces speibonae
1428652.BIV24_12925	84,4	244	38	0	6,2E-139	398	Streptomyces colonosanans
85558.T45_09073	81,3	251	47	0	4,1E-138	396	Streptomyces turgidiscabies
1428628.WN71_000320	82,8	250	43	0	5,8E-138	395	Streptomyces mangrovisoli
1890.AFM16_17520	81,7	251	46	0	8,3E-138	395	Streptomyces antibioticus
58343.AQJ46_18540	83,1	249	42	0	4,3E-137	394	Streptomyces canus
1155714.GCA_000373625_04709	82,4	250	44	0	4,8E-137	393	Streptomyces sp LaPpAH-108
68239.GCA_000745715_06200	83,1	249	42	0	2,5E-136	392	Streptomyces mirabilis
1428620.GCA_001005085_03371	81,9	249	45	0	2,8E-136	391	Streptomyces humi
33898.GCA_000772895_01513	82,4	244	43	0	3E-136	391	Streptomyces galbus
1915.SLINC_3770	84,4	243	38	0	7,7E-136	390	Streptomyces lincolnensis
1196353.SAMN05444921_11580	81,3	251	47	0	9,2E-135	387	Streptomyces wuyuanensis
1440053.GCA_000718095_02139	81,6	250	46	0	1,3E-134	387	Streptomyces scopuliridis RB72
680198.SCAB_51381	80,7	244	47	0	2E-134	387	Streptomyces scabiei 87 22
67304.AQJ54_36505	82,4	245	43	0	3,4E-134	386	Streptomyces griseorubiginosus
285458.BGM19_17330	81,2	250	47	0	7,5E-134	385	Streptomyces agglomeratus
1463921.GCA_000717945_04632	80,3	249	49	0	8,4E-134	385	Streptomyces sp NRRL S-920
83656.B1H18_30510	80	250	50	0	1,7E-133	384	Streptomyces tsukubensis
1169154.GCA_000377145_01849	81,6	250	46	0	2,2E-133	384	Streptomyces sp CNT372
1322334.A8W25_17310	80,4	250	49	0	3,1E-133	384	Streptomyces sp ERV7
545123.A7J05_20050	79,9	249	50	0	3,4E-133	384	Streptomyces silaceus
38300.SPRI_4220	81,2	250	47	0	4,4E-133	383	Streptomyces pristinaespiralis
67365.GCA_001704635_05547	80,5	251	49	0	4,4E-133	383	Streptomyces sparsogenes
591167.Sfla_2668	81,6	250	46	0	4,4E-133	383	Streptomyces pratensis ATCC 33331
1519471.ADK82_24930	81,2	250	47	0	4,4E-133	383	Streptomyces sp NRRL S-4
66429.GCA_000719265_05264	78,5	251	54	0	4,5E-133	383	Streptomyces roseoverticillatus
417292.SAMN05421806_12011	80,6	248	48	0	9,8E-133	382	Streptomyces indicus
862751.SACTE_3832	81,2	250	47	0	2,5E-132	381	Streptomyces sp SirexAA-E
1893.SAMN02787144_1007234	80	250	50	0	3,6E-132	381	Streptomyces atratus
1463820.GCA_000721375_03924	80,2	248	49	0	4E-132	381	Streptomyces sp NRRL B-3428
1736503.ASE41_22820	81,3	251	44	1	4,6E-132	380	Streptomyces sp Root264
67385.GCA_000725805_06229	80,4	250	49	0	5,1E-132	380	Streptomyces xanthophaeus
1736554.ASD51_16435	80	250	50	0	5,1E-132	380	Streptomyces sp Root55
1751294.ASR50_18960	79,9	249	50	0	5,1E-132	380	Streptomyces sp 4F
1463881.GCA_000716335_01975	79,2	250	52	0	7,2E-132	380	Streptomyces sp NRRL S-118
67267.GCA_000716675_05097	80,7	244	47	0	7,5E-132	381	Streptomyces alboflavus
67373.GCA_000718635_06058	81,1	244	46	0	9,3E-132	381	Streptomyces varsoviensis
1964449.GCA_002028385_03438	79,6	250	51	0	1E-131	380	Streptomyces sp 3211
1906740.BJP40_14625	79,7	251	51	0	1,5E-131	379	Streptomyces sp CC53
1157635.GCA_000373645_03486	79,6	250	51	0	2,1E-131	379	Streptomyces sp ATexAB-D23
1911.GCA_001715295_02445	80,4	250	49	0	2,9E-131	379	Streptomyces griseus
58346.BG653_05316	80,3	244	48	0	3,4E-131	379	Streptomyces platensis
1030533.GCA_001049855_03724	78,9	251	53	0	8,4E-131	377	Streptomyces sp Wb2n-11
1678637.AC230_14265	78,4	250	54	0	8,4E-131	377	Streptomyces caatingaensis
1703927.AMK16_24585	78,8	250	53	0	8,4E-131	377	Streptomyces sp CB00455
749414.SBI_04865	78,9	251	53	0	1,2E-130	377	Streptomyces bingchenggensis BCW-1
1172567.WQO_20380	79,6	250	51	0	1,7E-130	377	Streptomyces globisporus C-1027
1703920.AMK09_02330	78,4	250	54	0	1,7E-130	377	Streptomyces sp CB02488
1703937.AMK26_26310	79,6	250	51	0	1,7E-130	377	Streptomyces sp CB03234
1463920.GCA_000719335_05940	79,2	250	52	0	2,4E-130	376	Streptomyces sp NRRL S-87
465541.M444_15915	78,8	250	53	0	3,4E-130	376	Streptomyces sp Mg1
936756.ATE80_20425	78,5	251	54	0	3,4E-130	376	Streptomyces kanasensis
1894.ADK78_20065	78,7	244	52	0	5E-130	375	Kitasatospora aureofaciens
253839.SSNG_03940	78,4	250	54	0	6,9E-130	375	Streptomyces sp C
1206101.GCA_000515055_04652	77,3	251	57	0	6,9E-130	375	Streptomyces sp CNR698
193462.BBN63_19460	77,9	249	55	0	9,7E-130	375	Streptomyces niveus
1896.GCA_000718595_01468	78,1	251	55	0	1,4E-129	374	Streptomyces bikiniensis
1905.GCA_000725545_02503	78,4	250	54	0	1,4E-129	374	Streptomyces exfoliatus
1718998.A6A29_04380	78	250	55	0	1,4E-129	374	Streptomyces sp TSRI0281
1812480.B1K54_16280	78,8	250	53	0	1,4E-129	374	Streptomyces sp fd1-xmd
1718985.A6A06_05160	78,3	244	53	0	1,4E-129	374	Streptomyces sp CB02923
1855346.SAMN05216483_2693	77,6	250	56	0	2E-129	374	Streptomyces sp 2131 1
36818.BGK67_19075	78,8	250	53	0	2,8E-129	374	Streptomyces subrutilus
43759.GCA_000716445_07537	77,6	250	56	0	2,8E-129	374	Streptomyces wedmorensis
66430.ACS04_09815	80	250	50	0	4E-129	373	Streptomyces roseus
1054860.GCA_000384175_02859	79,2	250	52	0	4E-129	373	Streptomyces purpureus KA281
1463841.GCA_000720115_00377	78	250	55	0	4E-129	373	Streptomyces sp NRRL F-2580
1703933.AMK22_15515	78,8	250	53	0	4,1E-129	373	Streptomyces sp CB01580
1463900.GCA_000720175_07474	79,1	244	51	0	4,6E-129	373	Streptomyces sp NRRL S-337
1901.BB341_11605	78,1	251	55	0	8E-129	372	Streptomyces clavuligerus
1223523.H340_16076	76,4	250	59	0	8E-129	372	Streptomyces mobaraensis NBRC 13819 DSM 40847
47763.T261_3324	79,5	244	50	0	9,2E-129	372	Streptomyces lydicus

1881021.SAMN05428940_4469	78,7	244	52	0	9,2E-129	372	Streptomyces sp 2133 1
285500.GCA_001748305_05097	77,8	248	55	0	1,1E-128	372	Streptomyces luteocolor
68223.GCA_002028425_04027	78	250	55	0	1,1E-128	372	Streptomyces katrae
316284.SNOUR_17655	78,3	244	53	0	1,3E-128	372	Streptomyces noursei ATCC 11455
1343740.M271_28140	76,5	251	59	0	1,6E-128	372	Streptomyces rapamycinicus NRRL 5491
1048205.AB852_03915	79,4	243	50	0	1,9E-128	371	Streptomyces uncialis
1553907.GCA_000805335_04501	77,5	244	55	0	5,3E-128	370	Streptomyces sp CT34
1621259.GCA_001590865_00204	78,3	244	53	0	5,3E-128	370	Streptomyces sp NBRC 110611
1736450.ASD33_03290	78,6	248	53	0	5,3E-128	370	Streptomyces sp Root1304
75293.BV401_20480	75,3	251	62	0	6,5E-128	370	Streptomyces autolyticus
1935.B1H20_20525	78,4	250	54	0	9,3E-128	370	Streptomyces violaceoruber
1765722.AT728_04955	80,5	236	46	0	1,1E-127	369	Streptomyces silvensis
58344.GCA_000720995_06309	78,7	244	52	0	1,4E-127	369	Streptomyces celluloflavus
362257.SVTN_21860	77,6	250	56	0	1,9E-127	369	Streptomyces vietnamensis
1156844.GCA_000373565_00241	79,3	246	48	1	2,2E-127	369	Streptomyces sp HmicA12
42234.IQ63_11930	78	241	53	0	3,8E-127	368	Streptomyces acidiscabies
1576605.QR77_15580	77,5	249	56	0	3,8E-127	368	Streptomyces sp 150FB
68570.DC74_4861	77,5	244	55	0	6,2E-127	368	Streptomyces noursei
285473.A4G23_02340	78,5	251	54	0	7,6E-127	367	Streptomyces rubrolavendulae
1155718.GCA_000373585_04497	77	244	56	0	8,7E-127	367	Streptomyces sp MspMP-M5
66875.GCA_000718015_05456	76,2	244	58	0	1,6E-126	367	Streptomyces catenulae
1160718.SU9_11088	77,9	244	54	0	1,8E-126	367	Streptomyces auratus AGR0001
953739.SVEN_4208	77,8	248	55	0	2,5E-126	366	Streptomyces venezuelae ATCC 10712
1957.GCA_000720555_01333	77,9	244	54	0	3,3E-126	367	Streptomyces sclerotialus
1298880.GCA_000426165_04313	76,8	250	58	0	4,2E-126	365	Streptomyces sp TAA486
1286821.GCA_001418285_00292	76,4	250	59	0	6E-126	365	Streptomyces sp WMMB 322
66373.GCA_000718305_00986	77,9	244	54	0	2E-125	364	Streptomyces niger
68042.GCA_001553435_04048	75,7	251	61	0	5,1E-125	363	Streptomyces hygroscopicus subsp hygroscopicus
933944.AN215_09030	76,3	249	59	0	7E-125	362	Streptomyces abyssalis
996637.SGM_6117	78	245	54	0	8,6E-125	362	Streptomyces griseoaurantiacus M045
553510.B1H19_24860	76,2	248	58	1	1,1E-124	362	Streptomyces gilvosporeus
1306406.J116_015825	76,9	251	57	1	2E-124	361	Streptomyces thermolilacinus SPC6
310782.SAMN05216499_10733	76,2	244	58	0	2,2E-124	361	Actinacidiphila paucisporea
1134445.GCA_000258595_03185	76,1	251	60	0	2,9E-124	361	Streptomyces somaliensis DSM 40738
1609095.ADL22_27165	76,2	244	58	0	3,4E-124	361	Streptomyces sp NRRL F-4489
549819.GCA_001748085_03574	75,8	248	60	0	5,2E-124	360	Streptomyces sp TP-A0874
66377.GCA_000717745_02309	75,4	244	60	0	1,9E-123	359	Streptomyces violens
1463878.GCA_000725625_04209	76,7	245	57	0	2E-123	359	Streptomyces sp NRRL F-6676
1157637.GCA_000373665_03583	76,7	245	57	0	3,2E-123	358	Streptomyces sp BoleA5
1463854.GCA_000721445_03592	73,2	250	67	0	3,3E-123	358	Streptomyces sp NRRL F-5053
380248.SAMN05216251_1147	75,8	244	59	0	7,4E-123	357	Actinacidiphila alni
457427.SSOG_04955	74,9	251	63	0	9,8E-123	357	Streptomyces himastatinicus ATCC 53653
1305837.GCA_000426325_00644	76,1	243	58	0	1E-122	357	Streptomyces sp CNS606
403935.SAMN05216481_105264	76,4	246	58	0	1,2E-122	357	Streptomyces radiopugnans
1906.SFRA_28105	77,8	243	54	0	1,5E-122	357	Streptomyces fradiae
44060.GCA_000718985_03094	74,8	250	63	0	1,9E-122	356	Streptomyces megasporus
1463856.GCA_000721495_01479	75,6	242	59	0	4,3E-122	355	Streptomyces sp NRRL F-5123
1716141.STSP_45690	74,4	258	57	1	8,6E-122	355	Streptomyces jeddahensis
1288083.GCA_000424845_03183	74	250	65	0	4,6E-121	353	Streptomyces sp TAA040
1380346.GCA_000701325_02295	75,4	244	60	0	1E-120	352	Streptomyces sp URHA0041
1003195.SCATT_23810	74,2	244	63	0	2E-120	352	Streptantibioticus cattleyicolor NRRL 8057 DSM 46488
1888.Salbus254_2517	76,7	240	56	0	3E-120	351	Streptomyces albus
943816.AN217_04700	73,9	245	64	0	3,5E-120	351	Streptomyces qinglanensis
1380770.N566_01990	77	243	56	0	4,2E-120	350	Streptomycetaceae bacterium MP113-05
1463858.GCA_000719775_04311	72,7	249	68	0	3,8E-119	348	Streptomyces sp NRRL F-5135
1463857.GCA_000721265_00364	74,3	249	64	0	4,2E-119	348	Streptomyces sp NRRL F-5126
310780.SAMN05216267_1001146	73,1	242	65	0	1,3E-118	347	Actinacidiphila rubida
1288080.GCA_000424785_03281	73	244	66	0	1,4E-116	342	Streptomyces sp CNT360
1286822.GCA_000964305_05425	71,4	245	70	0	7E-114	335	Streptomyces sp WMMB 714
310781.SAMN05216259_106224	71,6	243	69	0	2E-113	333	Actinacidiphila guanduensis
1289387.GCA_000424965_00356	70,3	249	74	0	2,1E-112	331	Streptomyces sp TAA204
910347.SAMN05421773_102317	68,2	245	78	0	2,5E-111	329	Streptomyces aidingensis
1075402.AN216_04595	69	242	75	0	3,6E-110	325	Streptomyces oceani
1449355.GCA_000745345_02726	70,2	242	72	0	2,6E-109	324	Actinacidiphila yeochonensis CN732
1288082.GCA_000424825_00453	68,7	249	77	1	3,3E-109	323	Streptomyces sp CNH287
1550035.GCA_000974485_00411	66,1	251	85	0	5,6E-108	320	Streptomyces sp NBRC 109706
498367.GCA_001493375_00592	66,5	251	84	0	1,1E-105	314	Streptomyces specialis
1449353.GCA_000744815_03588	66,3	243	82	0	1,4E-105	313	Phaeacidiphilus oryzae TH49
1176198.SAMN05444716_10199	67,5	243	79	0	3,5E-105	313	Streptomyces harbinensis
76728.AQ490_17670	67,3	251	80	2	4E-105	312	Wenjunlia vitaminophila
408015.SXIM_32810	66,7	243	81	0	5,6E-105	313	Streptomyces xiamenensis
1857892.STBA_43880	66,9	251	83	0	7,1E-104	309	Streptomyces sp MP131-18
1736486.ASE03_02030	64,9	242	85	0	7,7E-103	306	Kitasatospora sp Root187
1123321.GCA_000381025_05939	66	250	85	0	7,9E-103	306	Streptomyces sulphureus DSM 40104
105420.GCA_000787815_06409	65,4	243	84	0	2,2E-102	305	Streptacidiphilus neutrinimicus
**235985.SAMN05414137_10195**	65,8	243	83	0	3,1E-102	305	Streptacidiphilus jiangxiensis
981369.GCA_000744655_08178	64,2	243	87	0	3,6E-101	302	Streptacidiphilus rugosus AM-16
1449352.GCA_000744705_03685	63,4	243	89	0	5,1E-101	302	Streptacidiphilus albus JL83
1804758.A6A07_32195	64,5	242	86	0	7,2E-101	301	Streptomyces sp CB03911
2064.TR51_31775	64,5	242	86	0	2,1E-100	300	Kitasatospora griseola

1449347.GCA_000744225_03651	64,6	246	82	1	9,9E-100	299	Kitasatospora mediocidica KCTC 9733
1463887.GCA_000722215_01279	64,3	241	86	0	1,38E-98	296	Streptomyces sp NRRL S-1777
500153.GCA_000719135_02747	64,1	245	88	0	2,24E-98	295	Streptomyces avicenniae
1449346.GCA_000744785_04062	62,4	242	91	0	7,91E-98	294	Kitasatospora azatica KCTC 9699
452652.KSE_44780	62,8	242	90	0	1,12E-97	293	Kitasatospora setae KM-6054
1463833.GCA_000717715_02871	62,8	242	90	0	4,55E-97	292	Streptomyces sp NRRL B-24484
1463902.GCA_000719705_02073	62,8	247	87	1	1,02E-94	286	Streptomyces sp NRRL S-350
1428644.BIV57_03990	61,4	246	93	1	3,74E-94	285	Mangrovactinospora gilvigrisea
68260.GCA_000717725_03057	61,5	247	90	1	4,78E-93	282	Streptomyces pyridomyceticus
67352.GCA_000718025_02598	61,1	247	91	1	7,83E-92	279	Kitasatospora purpeofusca
591157.SSLG_02719	69,1	207	64	0	1,26E-90	274	Streptomyces sp SPB78
1120523.GCA_001625365_02926	59,5	247	95	1	5,19E-90	274	Streptomyces sp MJM8645
1463934.GCA_000720635_00318	58,7	247	97	1	2,42E-88	270	Streptomyces sp NRRL WC-3742
