# Supplementary material for: The novel GlcNAc 6-phosphate dehydratase NagS governs a metabolic checkpoint that controls nutrient signaling in Streptomyces
Source: PLoS Biol. 2025 Nov 25;23(11):e3003514. doi: 10.1371/journal.pbio.3003514 (PMC12680351; doi:10.1371/journal.pbio.3003514)
Supplement: S5 Table — (PDF) [file pbio.3003514.s019.pdf]

S5 Table. NMR data of 1 as compared to Chromogen I [9]

Position		δ_H, mult. (J in Hz)		δ_C, type	
		**1**[a]	Chromogen I[b]	**1**[c]	Chromogen I[d]
α-anomer[e]	1	6.06, dd (4.0, 1.0)	6.01, dd (4.0, 0.9)	102.1, CH	102.4, CH
	2			ND	137.3, C
	3	6.19, dd (1.7, 1.0)	6.14, br s	110.4, CH	112.2, CH
	4	5.10, td (4.0, 1.7)	5.03, td (4.0, 1.6)	87.7, CH	87.9, CH
	5	3.89, m	3.80, td (4.0, 7.2)	75.6, CH	76.4, CH
	6	a: 3.88, m<br>b: 3.79, m	a: 3.69, dd (11.9, 4.0)<br>b: 3.55, dd (11.9, 7.2)	67.8, CH_2	65.4, CH_2
	CH_3 (*N*-acetyl)	2.11, s	2.10, s	25.4, CH_3	25.6, CH_3
	CO (*N*-acetyl)			176.2, C	176.5, C
β-anomer	1	5.99, t (1.0)	5.97, d (1.0)	102.0, CH	102.3, CH
	2			ND	136.8, C
	3	6.22, dd (1.4, 1.0)	6.20, dd (1.6, 1.0)	113.3, CH	113.1, CH
	4	4.88, m	4.81, d (1.6)	87.2, CH	87.5, CH
	5	3.81	3.75–3.71, m	76.0, CH	76.8, CH
	6	3.93, m	a: 3.75–3.71, m<br>b: 3.64–3.59, m	67.9, CH_2	65.5, CH_2
	CH_3 (*N*-acetyl)	2.11, s	2.10, s	25.4, CH_3	25.6, CH_3
	CO (*N*-acetyl)			176.2, C	176.5, C

[a] 600 MHz at 298 K in D_2O, [b] 500 MHz in D_2O. Temperature is not given, [c] 213 MHz at 298 K in D_2O, [d] 125 MHz in D_2O. Temperature is not given, [e] Ratio of the anomers α:β is 1:0.6 for both **1** and chromogen I, ND: Not detectable
