# Supplementary material for: The novel GlcNAc 6-phosphate dehydratase NagS governs a metabolic checkpoint that controls nutrient signaling in Streptomyces
Source: PLoS Biol. 2025 Nov 25;23(11):e3003514. doi: 10.1371/journal.pbio.3003514 (PMC12680351; doi:10.1371/journal.pbio.3003514)
Supplement: S6 Table — (PDF) [file pbio.3003514.s020.pdf]

S6 Table. HRMS data of the compounds identified in this study

Compounds	Molecular formula	Calculated $[M-H]^-$ *m/z*	Observed *m/z*
**1**	$C_8H_{14}NO_8P$	282.0384	282.0387
**2/3**	$C_6H_{12}NO_7P$	240.0279	240.0280
