# Supplementary material for: The novel GlcNAc 6-phosphate dehydratase NagS governs a metabolic checkpoint that controls nutrient signaling in Streptomyces
Source: PLoS Biol. 2025 Nov 25;23(11):e3003514. doi: 10.1371/journal.pbio.3003514 (PMC12680351; doi:10.1371/journal.pbio.3003514)
Supplement: S7 Table — (PDF) [file pbio.3003514.s021.pdf]

S7 Table. Molecular weight standards used for size-exclusion chromatography calibration

Protein	Molecular Weight (M_w, KDa)	Elution Volume (V_e, mL)
Thyroglobulin bovine	670	10.11
γ-globulins from bovine blood	150	12.39
Ovalbumin	44.3	15.44
Ribonuclease A type I-A	13.7	18.08
p-aminobenzoic acid (pABA)	0.14	22.94
